# Platelets Orchestrate a Neuroimmune Axis Driving Cutaneous Inflammation and Itch

**DOI:** 10.34133/research.1294

**Published:** 2026-05-28

**Authors:** Ximin Hu, Fujun Wang, Ting Wang, Yifei Liu, Lei Zhang, Libei Liu, Liang Cao, Shaofeng Pu, Ronghua Yang, Jing Feng

**Affiliations:** ^1^ Department of Burn and Plastic Surgery, School of Medicine, the Second Affiliated Hospital of South China University of Technology (Guangzhou First People’s Hospital), Guangzhou 510180, China.; ^2^ State Key Laboratory of Chemical Biology, Shanghai Institute of Materia Medica, Shanghai 200000, China.; ^3^ Department of Dermatology, Xiangya Hospital, Central South University, Changsha, Hunan 410000, China.; ^4^ School of Basic Medicine, Qiqihar Medical University, Qiqihar, Heilongjiang 161006, China.; ^5^ Department of Chinese Medicine, The Second Affiliated Hospital of Air Force Medical University, Xi’an, Shaanxi 710038, China.; ^6^ Department of Pain Management, Shanghai Jiao Tong University Affiliated Sixth People’s Hospital, 200000, Shanghai, China.; ^7^ University of Chinese Academy of Sciences, Beijing 100000, China.

## Abstract

Platelets are traditionally recognized for their roles in hemostasis, but their involvement as active immune modulators in cutaneous neuroimmune signaling remains poorly understood. In this study, we integrated multi-modal genetic and pharmacological strategies to investigate the functional contribution of platelets to skin inflammation and chronic itch. Optogenetic activation of platelets was sufficient to elicit localized skin inflammation, erythema, and robust pruritus, with transcriptomic profiling of lesions showing strong concordance with the clinical signatures of atopic dermatitis (AD). In experimental AD models, platelet depletion markedly reduced scratching behavior, inflammatory cell infiltration, and C-fiber excitability, whereas platelet activation exacerbated these phenotypes. Mechanistically, activated platelets release serotonin [5-hydroxytryptamine (5-HT)], which compromises vascular integrity and facilitates platelet extravasation into the dermis. This “neuroimmune hub” promotes macrophage recruitment and sensitizes *TRPV1^+^* pruriceptors. Transcriptomic analysis revealed that platelet-derived 5-HT drives these processes via HTR2B and HTR7 signaling. Specifically, genetic ablation of *HTR2B* in *TRPV1^+^* neurons selectively impaired itch transmission. Furthermore, re-analysis of clinical datasets confirmed the enrichment of *HTR2B* and *HTR7* in skin macrophage populations during inflammation. Systemic administration of HTR antagonists or the anti-platelet agent clopidogrel markedly attenuated both inflammation and pruritus across multiple models. Our findings identify the platelet–immune–neuron axis as a key driver of cutaneous dysfunction and a promising therapeutic target for chronic inflammatory skin disorders.

## Introduction

Platelets have long been defined by their classical functions in hemostasis and thrombosis [1,2]. However, a paradigm shift in modern immunology has repositioned these anucleate fragments as sophisticated immune orchestrators capable of sensing pathogen-associated molecular patterns (PAMPs) and danger-associated molecular patterns (DAMPs) via an extensive repertoire of surface receptors [3–5]. Beyond their systemic roles, a growing body of clinical evidence links platelet dysfunction to the pathogenesis of chronic inflammatory skin diseases. Patients with atopic dermatitis (AD) and psoriasis consistently exhibit systemic platelet activation, characterized by elevated plasma levels of β-thromboglobulin and platelet factor 4 (PF4), markers that directly correlate with disease severity [6]. Furthermore, increased circulating platelet–leukocyte aggregates and microparticles in AD patients suggest a state of chronic priming that precedes tissue-level inflammation [7]. Despite these clinical associations [8,9], whether platelet activation is a causal driver or a secondary consequence of chronic skin inflammation and itch remains poorly understood.

Within the skin, platelets engage in intimate crosstalk with immune cells and sensory neurons [10,11]. Platelets accumulate at inflamed skin sites and release bioactive mediators that can amplify immune cell infiltration and modulate peripheral neural excitability [10,12–14]. However, dissecting the precise functional contribution of platelets in vivo has remained technically elusive. Pharmacological depletion and systemic knockout approaches often confound results with global physiological perturbations. Recently, Zhang et al. [15] introduced second-generation channelrhodopsin constructs into megakaryocytes and platelets, highlighting the power of optogenetics in dissecting platelet biology. Building on this approach, we utilized a *PF4^Cre^; Ai32* system to achieve spatiotemporal control over platelet activation in the skin. This approach allows us to bypass systemic triggers and directly test the “platelet-first” hypothesis in skin inflammation.

Among the various mediators, 5-hydroxytryptamine (5-HT) serves as a critical candidate for bridging the immune–nerve interface. Stored at exceptionally high concentrations within platelet-dense granules, 5-HT regulates vascular permeability, leukocyte recruitment, and sensory transduction [16–18]. In AD, cutaneous 5-HT levels are markedly elevated and intradermal administration of 5-HT is sufficient to evoke erythema, edema, and robust pruritus [19–21]. While 5-HT receptor signaling, particularly via HTR2B and HTR7, has been implicated in inflammation, the specific cellular source of 5-HT and its direct targets on sensory neurons or other immune cells during chronic dermatitis remain poorly defined [22].

In this study, we demonstrate that selective optogenetic activation of platelets is sufficient to initiate a localized inflammatory response and pruritic behavior that pathogenetically resemble human AD. By combining platelet depletion, serotonin genetic ablation, and neuron-specific receptor deletion, we uncover a previously unrecognized platelet–immune–neuron axis. We identify platelet-derived 5-HT as the central mediator that drives macrophage recruitment via HTR2B and HTR7, and directly sensitizes *TRPV1^+^* pruriceptors via HTR2B. Our findings establish platelets as essential drivers of neuroimmune amplification, positioning them and their downstream signaling pathways as high-value therapeutic targets for the management of AD and other chronic pruritic disorders.

## Results

### Optogenetic activation of platelets is sufficient to drive chronic itch and skin inflammation

**Fig. 1.**
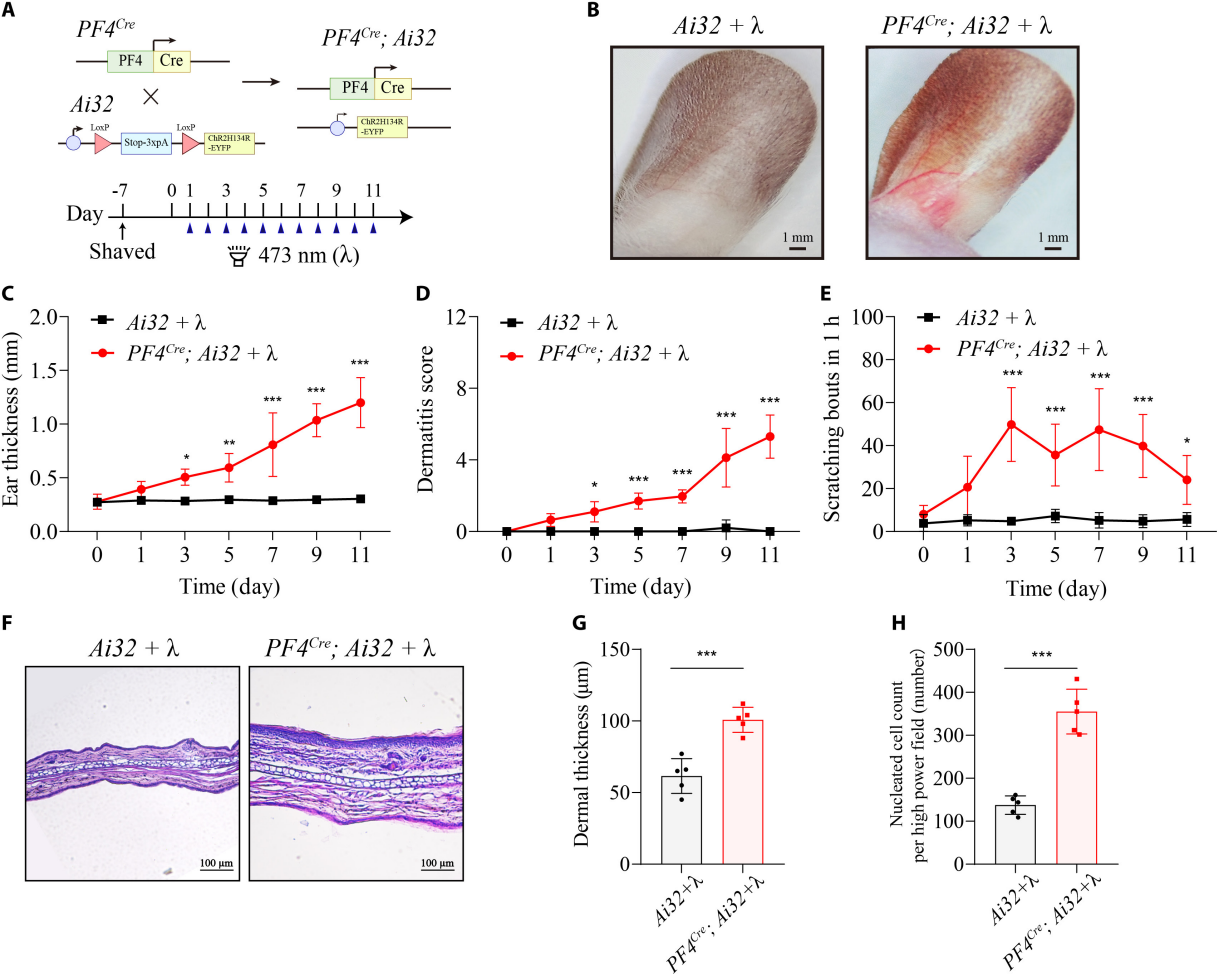
Optogenetic platelet activation elicits cutaneous inflammatory responses and pruritic behavior. (A) Schematic illustration of the generation of *PF4^Cre^; Ai32* mice enabling optogenetic platelet activation. (B) Representative images of ears from *Ai32* and *PF4^Cre^; Ai32* mice after 11 d of photostimulation (+λ). Scale bar, 1 mm. (C and D) Ear thickness (C) and inflammation score (D) measured at the indicated time points during photostimulation. *n* = 5 mice per group. For (C), *F*_6,56_ = 19.01, *P* < 0.0001. Day 3: *P* = 0.0353, day 5: *P* = 0.0018, day 7: *P* < 0.0001, day 9: *P* < 0.0001, day 11: *P* < 0.0001. For (D), *F*_6,56_ = 24.32, *P* < 0.0001. Day 3: *P* = 0.0386, day 5: *P* = 0.0003, day 7: *P* = 0.0022, day 9: *P* < 0.0001, day 11: *P* < 0.0001 (2-way ANOVA followed by Šídák’s multiple comparisons test). (E) Quantification of spontaneous scratching bouts in photostimulated *Ai32* and *PF4^Cre^; Ai32* mice. *n* = 5 mice per group. *F*_6,56_ = 5.246, *P* = 0.0002. Day 3: *P* < 0.0001, day 5: *P* = 0.0004, day 7: *P* < 0.0001, day 9: *P* < 0.0001, day 11: *P* = 0.0467 (2-way ANOVA followed by Šídák’s multiple comparisons test). (F) Representative H&E histopathology images of ear sections from photostimulated *Ai32* and *PF4^Cre^; Ai32* mice. *n* = 3 to 5 sections from 3 mice. Scale bar, 100 μm. (G and H) Quantification of dermal thickness (G) and dermal immune cell infiltration (H). *n* = 5 mice per group. For (G), *t*_8_ = 5.857, *P* = 0.0004. For (H), *t*_8_ = 8.624, *P* < 0.0001 (2-tailed unpaired Student’s *t* test). *P < 0.05, **P < 0.01, ***P < 0.001. Individual data points in the remaining panels represent single animals and are shown as mean ± SD from one representative of 3 independent experiments with consistent results.

To address whether platelets are sufficient to induce chronic itch and skin inflammation, we established *PF4^Cre^; Ai32* mice and performed optogenetics to activate platelets precisely in temporal and spatial precision (Fig. 1A). *PF4^Cre^; Ai32* mice were used to achieve platelet-specific expression of ChR2-YFP (yellow fluorescent protein). YFP-positive platelets were visualized by immunofluorescence microscopy and flow cytometry (Fig. S1). Baseline platelet activation, scratching behavior, and skin morphology were unchanged between *Ai32* and *PF4^Cre^; Ai32* mice (Figs. S2 and S3). Optogenetic stimulation significantly enhanced platelet activation (Fig. S4) and induced erythema, increased ear thickness, and robust scratching behavior (Fig. 1B to E). Histology following 11 d of photostimulation revealed dense immune cell infiltration and keratinocyte proliferation (Fig. 1F to H). In contrast, clopidogrel treatment (a platelet function inhibitor targeted to the platelet adenosine diphosphate receptor P2Y12) markedly attenuated platelet photoactivation-induced scratching behavior and significantly reduced the skin inflammatory response (Fig. S5). Our findings establish that platelet activation alone is adequate to initiate chronic itch and skin inflammation.

### Activation of platelets drives itch transmission and orchestrates macrophage recruitment

**Fig. 2.**
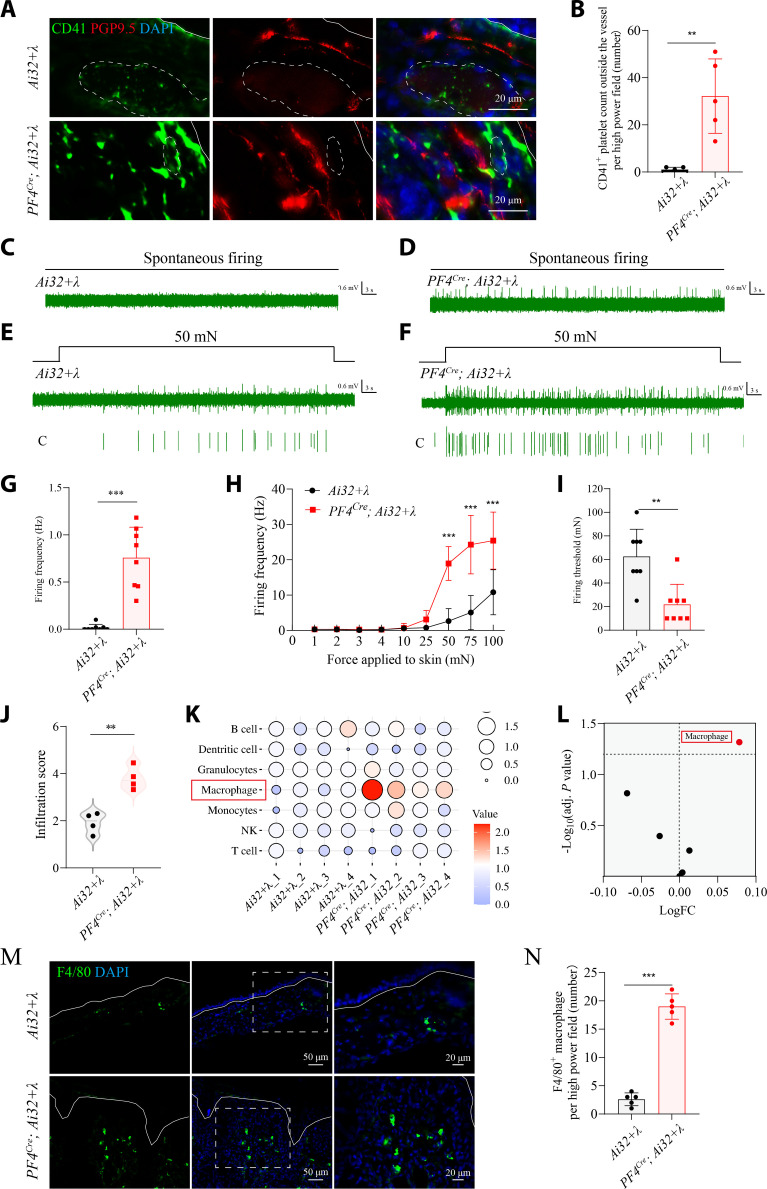
Platelet activation is sufficient to enhance C-fiber excitability and macrophage infiltration. (A) Representative images of CD41^+^ platelets (green) and PGP9.5^+^ nerve fibers (red) in the skin of photostimulated *Ai32* and *PF4^Cre^; Ai32* mice. Solid line delineates the dermal–epidermal junction, and dashed line outlines blood vessels. Scale bar, 20 μm. (B) Quantification of extravascular CD41^+^ platelets. *n* = 5 mice per group. *t*_8_ = 4.415, *P* = 0.0022 (2-tailed unpaired Student’s *t* test). (C and D) Representative traces of spontaneous C-fiber firings recorded ex vivo from the photostimulated *Ai32* (C) and *PF4^Cre^; Ai32* mice (D). (E and F) Representative traces of mechanically evoked C-fiber firings in response to 50-mN stimulation from the photostimulated *Ai32* (E) and *PF4^Cre^; Ai32* mice (F). (G to I) Summarized data showing spontaneous C-fiber firing frequency (G), mechanically evoked C-fiber firing frequency (H), and mechanical firing threshold (I). *n* = 8 units from 5 mice. For (G), *P* = 0.0002 (2-tailed Mann–Whitney test). For (H), *F*_8,126_ = 20.71, *P* < 0.0001. 50 mN: *P* < 0.0001, 75 mN: *P* < 0.0001, 100 mN: *P* < 0.0001 (2-way ANOVA followed by Šídák’s multiple comparisons test). For (I), *P* = 0.0026 (2-tailed Mann–Whitney test). (J) *ImmuCellAI* analysis scores of the immune infiltration based on RNA-seq data. *n* = 4 mice per group. *t_6_* = 5.736, *P* = 0.0012 (2-tailed unpaired Student’s *t* test). (K and L) Bubble plot (K) and volcano plot (L) showed an overview of adjusted scores for kinds of immune cells. For (L), macrophage: logFC = 0.078, −log_10_(adj. *P* value) = 1.318. (M and N) Representative images of F4/80 macrophages in the skin of photostimulated *Ai32* and *PF4^Cre^; Ai32* mice, which were quantified in (N). Scale bar, as indicated. *n* = 5 mice per group. For (N), *t*_8_ = 14.61, *P* < 0.0001 (2-tailed unpaired Student’s *t* test). *P < 0.05, **P < 0.01, ***P < 0.001. Individual data points in (G) to (I) represent single animals, and bars show mean ± SD from 2 independent experiments. Result in (B) and (N), individual data points in the remaining panels represent single animals and are shown as mean ± SD from one representative of 2 independent experiments with consistent results.

To delineate how platelet activation contributes to neural and immune responses in the skin, we first performed high-resolution immunofluorescence imaging of *PF4^Cre^; Ai32* mice. In photostimulated *Ai32* controls, CD41^+^ platelets were confined to the vascular lumen, consistent with their normal intravascular distribution (Fig. 2A and B). In contrast, photostimulated *PF4^Cre^; Ai32* mice exhibited a robust extravascular accumulation of CD41^+^ platelets in the dermis (Fig. 2A and B). Furthermore, Evans blue staining revealed a marked increase in dye extravasation after blue light stimulation, indicating enhanced vascular permeability (Fig. S6A to C). Consistently, CD41 immunofluorescence staining showed an increase in vascular diameter in the stimulated tissue (Fig. S6D to H). Together, these findings demonstrate that platelet activation promotes local vascular leakage and vasodilation, which might aggravate the platelet infiltration.

Moreover, infiltrated CD41^+^ platelets closely aligned with densely innervating PGP9.5^+^ sensory fibers in photostimulated lesions (Fig. 2A and B). This precise spatial juxtaposition suggested that activated platelets may engage in direct neuroimmune interactions capable of altering sensory neuron activity. To test the functional relevance of these structural observations, we performed ex vivo skin–nerve recordings. Nerve fibers from photostimulated *PF4^Cre^; Ai32* mice exhibited markedly elevated spontaneous firing (Fig. 2C, D, and G) and enhanced mechanically (Fig. 2E, F, H, and I) evoked responses compared to controls, demonstrating that platelet activation directly heightens peripheral sensory excitability, a key feature of itch sensitization.

To further characterize the immunological consequences of platelet activation, we conducted bulk RNA-sequencing (RNA-seq) analysis of photostimulated skin. Gene ontology (GO) enrichment revealed pronounced up-regulation of inflammatory and sensory neuron-related pathways, including cytokine signaling, leukocyte chemotaxis, and neuroactive ligand–receptor interactions (Figs. S7 and S8). Computational immune deconvolution using *ImmuCellAI* analysis indicated that macrophages were the dominant infiltrating population following platelet activation (Fig. 2J to L). Consistently, immunohistological staining confirmed dense macrophage accumulation throughout the dermis of photostimulated *PF4^Cre^; Ai32* mice (Fig. 2M and N). Together, these results demonstrate that activated platelets are not passive bystanders in the skin but potent initiators of both neural sensitization and immune cell recruitment.

### Platelet infiltration is required for itch transmission and macrophage accumulation in AD

**Fig. 3.**
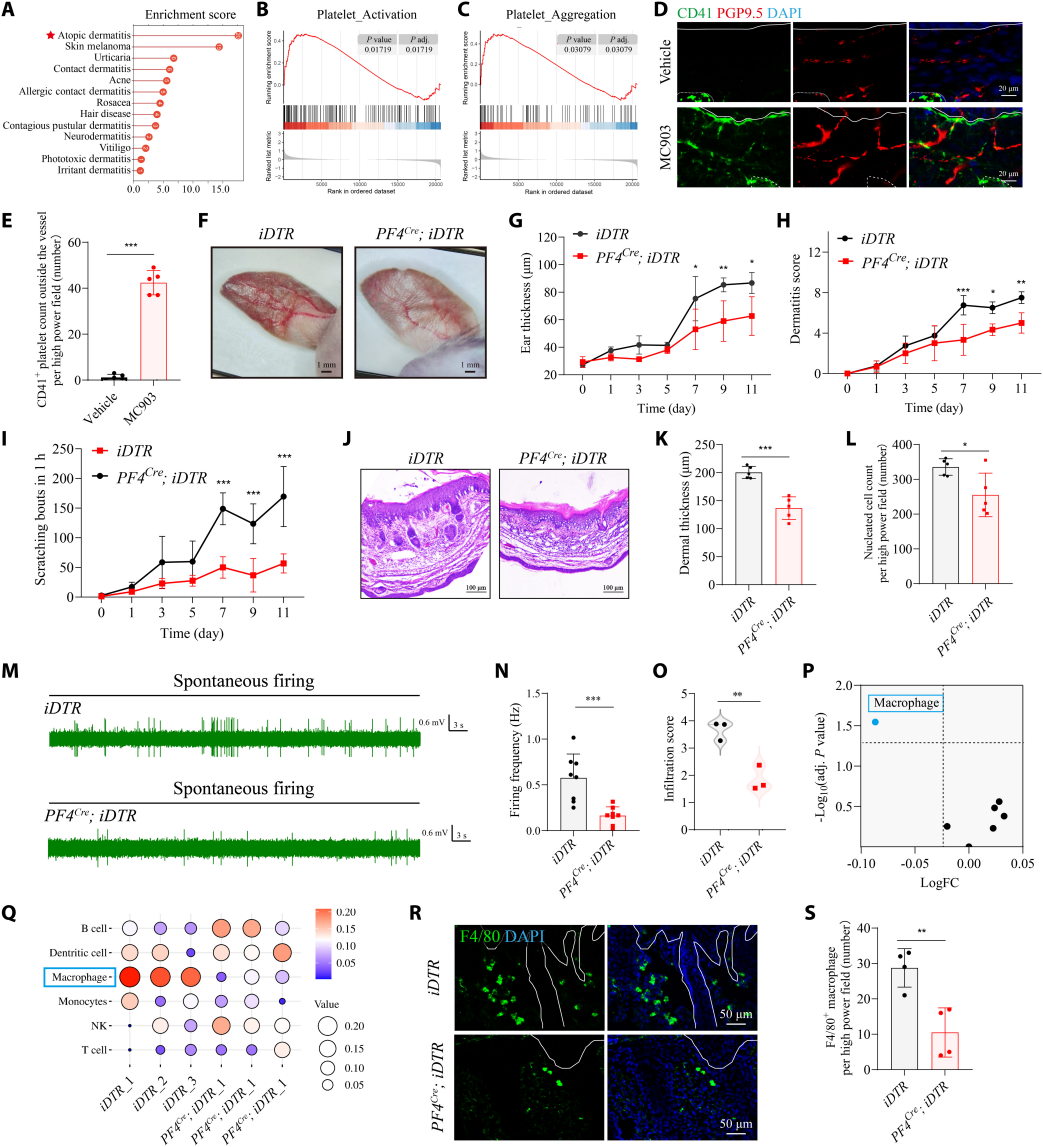
Platelet infiltration is necessary for itch transmission and macrophage accumulation in AD. (A) Pathogenetic resemblance analysis between photostimulated *PF4^Cre^; Ai32* mice and human cutaneous diseases using the *GeneCards* platform. (B and C) GSEA analysis of the platelet activation (B) and aggregation (C) pathways in AD patients. *n* = 53 healthy individuals, *n* = 59 AD patients (GSE99802). For (B), *P* = 0.01719. For (C), *P* = 0.03079. (D) Representative images of CD41^+^ platelets (green) and PGP9.5^+^ nerve fibers (red) in the skin of MC903-induced AD mouse model. Scale bar, 20 μm. Solid line delineates the dermal–epidermal junction, and dashed line outlines the vasculature. (E) Quantification of extravascular CD41^+^ platelets. *n* = 5 mice per group. *t*_8_ = 16.96, *P* < 0.0001 (2-tailed unpaired Student’s *t* test). (F) Representative images of ears from platelet-depleted *PF4^Cre^; iDTR* mice and control littermates after MC903 treatment. Scale bar, 1 mm. (G and H) Ear thickness (G) and inflammation score (H) measured at the indicated time points. For (G), *F*_6,56_ = 6.233, *P* < 0.0001. Day 7: *P* = 0.0276, day 9: *P* = 0.0065, day 11: *P* = 0.0153. For (H), *F*_6,56_ = 5.896, *P* = 0.0047. Day 7: *P* < 0.0001, day 9: *P* = 0.0187, day 11: *P* = 0.0048 (2-way ANOVA followed by Šídák’s multiple comparisons test). (I) Quantification of spontaneous scratching bouts in platelet-depleted *PF4^Cre^; iDTR* mice and the control littermates after MC903 treatment. *n* = 5 mice per group. *F*_6,56_ = 7.772, *P* < 0.0001. Day 7: *P* < 0.0001, day 9: *P* < 0.0001, day 11: *P* < 0.0001 (2-way ANOVA followed by Šídák’s multiple comparisons test). (J) Representative H&E histopathology images of ear sections. *n* = 3 to 5 sections from 3 mice. Scale bar, 100 μm. (K and L) Quantification of dermal thickness (K) and dermal immune cell infiltration (L). *n* = 5 mice per group. For (K), *t*_8_ = 6.223, *P* = 0.0003. For (L), *t*_8_ = 2.686, *P* = 0.0277 (2-tailed unpaired Student’s *t* test). (M) Representative traces of spontaneous C-fiber firings recorded ex vivo from platelet-depleted *PF4^Cre^; iDTR* mice (top) and the control littermates (bottom) after MC903 treatment. (N) Summarized data showing spontaneous C-fiber firing frequency. *n* = 8 units from 4 mice. *t_14_* = 4.212, *P* = 0.0009 (2-tailed unpaired Student’s *t* test). (O) *ImmuCellAI* analysis scores of the immune infiltration based on RNA-seq data. *n* = 3 mice per group. *t_4_* = 5.485, *P* = 0.0054 (2-tailed unpaired Student’s *t* test). (P and Q) Volcano plot (P) and bubble plot (Q) showed an overview of adjusted scores for kinds of immune cells. For (Q), macrophage: logFC = −0.0868, −log_10_(adj. *P* value) = 1.545. (R and S) Representative images of F4/80 macrophages in the skin of MC903-treated *PF4^Cre^; iDTR* mice and the control littermates, which were quantified in (S). Scale bar, 50 μm. *n* = 4 mice per group. For (S), *t_6_* = 4.135, *P* = 0.0061 (2-tailed unpaired Student’s *t* test). *P < 0.05, **P < 0.01, ***P < 0.001. Individual data points in (N) represent single animals, and bars show mean ± SD from 2 independent experiments. Result in (E), (G) to (I), (K), (L), and (S), individual data points represent single animals and are shown as mean ± SD from one representative of 2 independent experiments with consistent results.

Because photostimulated lesions exhibited inflammatory and transcriptomic features reminiscent of human AD, we next examined whether platelet activation causally drives disease-associated itch and inflammation. Differential expression analysis comparing our platelet-driven transcriptome with human disease signatures revealed strong similarity to AD (Fig. 3A). Notably, gene set enrichment analysis (GSEA) of patient datasets showed marked up-regulation of platelet activation and aggregation pathways in AD skin relative to healthy controls (Fig. 3B and C). Further reanalysis of a spatial transcriptomic dataset (GSE197023) showed the enriched expression of platelet-associated genes (*CLIC1*, *CTSG*, *EMILIN1*, *EMILIN2*, and *HSPB1*) specifically within the dermal compartment of AD lesions (Fig. S9). Immunohistology confirmed robust CD41^+^ platelet infiltration in MC903-induced AD (Fig. 3D and E). Flow cytometric analysis revealed a modest up-regulation of CD62P expression in circulating blood platelets from MC903-induced AD mice (Fig. S10), reinforcing the pathological relevance of platelet recruitment and activation in chronic dermatitis. In AD lesions, Evans blue staining and CD41 immunofluorescence staining showed an increase in vascular permeability and vascular diameter, which may promote platelet infiltration (Fig. S11).

To determine whether platelets are mechanistically required for AD-associated itch and inflammation, we first performed correlation analysis and revealed a positive relation between platelet and inflammation or itch process in AD (Fig. S12). To further assess functional relevance of platelets, we selectively depleted platelets in *PF4^Cre^; iDTR* mice using diphtheria toxin (DTX) (Fig. S13). Platelet-depleted mice exhibited normal skin morphology and behavior at baseline (Fig. S14), confirming that acute depletion alone does not perturb homeostasis. However, when subjected to MC903 treatment, DTX-treated mice showed markedly reduced ear swelling, dermal thickening, inflammatory cell infiltration, and spontaneous scratching (Fig. 3F to L). Consistently, clopidogrel treatment similarly alleviated chronic itch and skin inflammation in MC903-induced AD mice (Fig. S15).

Electrophysiological recordings and immunohistology further revealed that C-fiber excitability and macrophage infiltration, normally heightened in AD, were significantly suppressed following platelet depletion (Fig. 3M to S and Fig. S16). In contrast, optogenetic hyperactivation of platelets exacerbated inflammation and pruritus (Fig. S17), macrophage recruitment (Fig. S18), and C-fiber hyperexcitability (Fig. S19) in MC903-treated animals. These results establish platelets as indispensable drivers of neuroimmune dysfunction in AD.

### Platelet-derived serotonin is a key mediator linking platelet activation to itch and macrophage infiltration

**Fig. 4.**
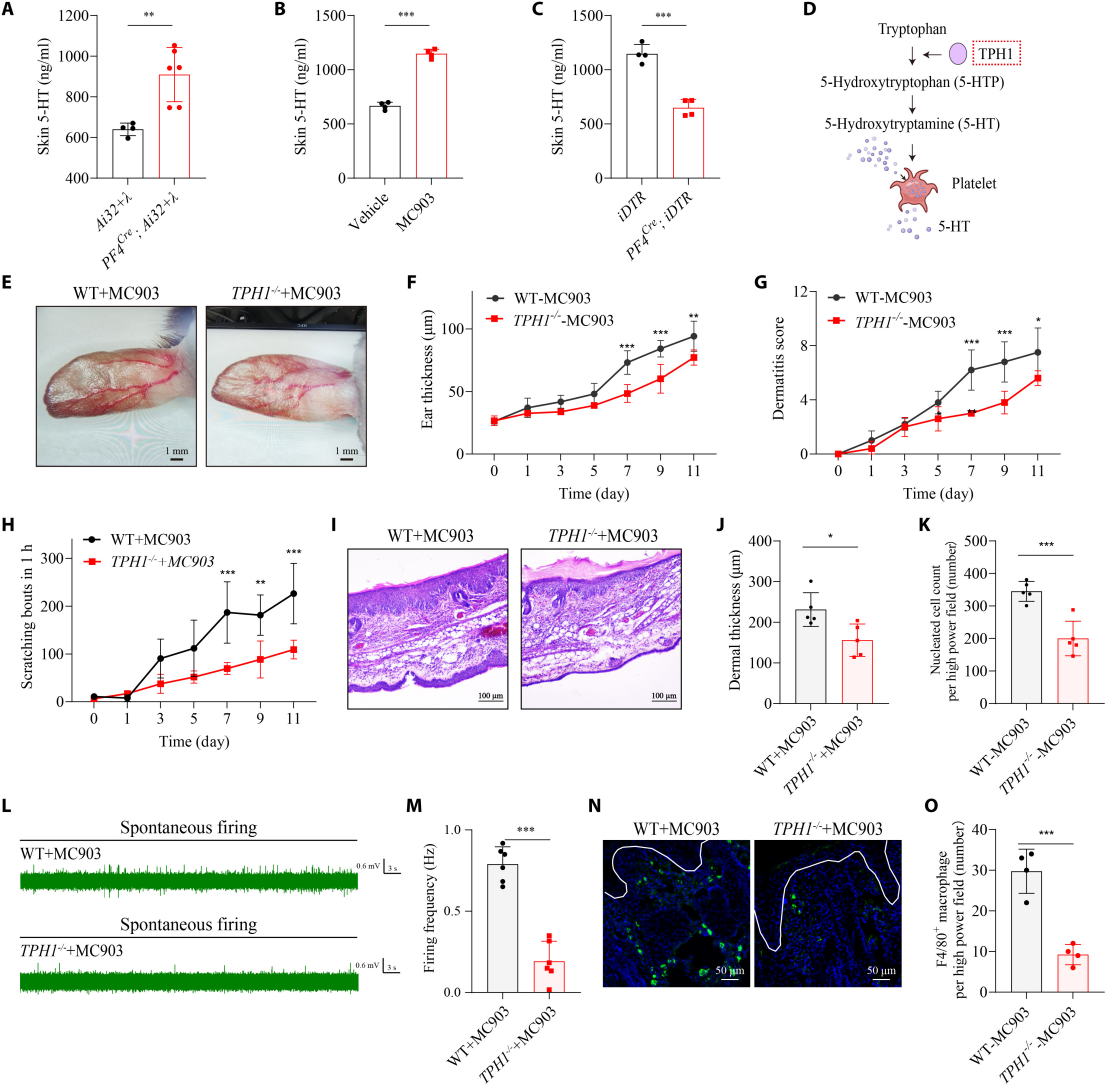
Platelet-derived serotonin contributes to itch transmission and macrophage accumulation in AD. (A) Cutaneous 5-HT concentrations measured by ELISA in photostimulated *Ai32* and *PF4^Cre^; Ai32* mice. *n* = 4 photostimulated *Ai32* mice. *n* = 6 photostimulated *PF4^Cre^; Ai32* mice. *t*_8_ = 3.900, *P* = 0.0045 (2-tailed unpaired Student’s *t* test). (B) Skin 5-HT concentrations measured by ELISA in the vehicle- and MC903-treated mice. *n* = 4 mice per group. *t_6_* = 17.93, *P* < 0.0001 (2-tailed unpaired Student’s *t* test). (C) Skin 5-HT concentrations measured by ELISA in the platelet-depleted *PF4^Cre^; iDTR* mice and control littermates after MC903 treatment. *n* = 4 mice per group. *t_6_* = 8.558, *P* = 0.0001 (2-tailed unpaired Student’s *t* test). (D) Schematic drawing of peripheral 5-HT synthesis and release pathway. (E) Representative images of ears from WT and *TPH1^−/−^* after MC903 treatment. Scale bar, 1 mm. (F and G) Ear thickness (F) and inflammation score (G) measured at the indicated time points. For (F), *F*_6,56_ = 4.830, *P* = 0.0005. Day 7: *P* < 0.0001, day 9: *P* < 0.0001, day 11: *P* = 0.0022. For (G), *F*_6,56_ = 5.066, *P* = 0.0003. Day 7: *P* < 0.0001, day 9: *P* < 0.0001, day 11: *P* = 0.0120 (2-way ANOVA followed by Šídák’s multiple comparisons test). (H) Quantification of spontaneous scratching bouts in WT and *TPH1^−/−^* after MC903 treatment. *n* = 5 mice per group. *F*_6,56_ = 3.936, *P* = 0.0026. Day 7: *P* < 0.0001, day 9: *P* = 0.0017, day 11: *P* < 0.0001 (2-way ANOVA followed by Šídák’s multiple comparisons test). (I) Representative H&E histopathology images of ears. *n* = 3 to 5 sections from 3 mice. Scale bar, 100 μm. (J and K) Quantification of dermal thickness (J) and dermal immune cell infiltration (K). *n* = 5 mice per group. For (J), *t*_8_ = 2.935, *P* = 0.0188. For (K), *t*_8_ = 5.305, *P* = 0.0007 (2-tailed unpaired Student’s *t* test). (L and M) Representative traces of spontaneous C-fiber firings recorded ex vivo, quantified in (M). *n* = 6 units from 4 mice. *t_10_* = 8.941, *P* < 0.0001 (2-tailed unpaired Student’s *t* test). (N and O) Representative images of F4/80^+^ macrophages in the skin of MC903-treated WT and *TPH1^−/−^* mice, which were quantified in (O). Scale bar, 50 μm. *n* = 4 mice per group. For (O), *t_6_* = 6.849, *P* = 0.0005 (2-tailed unpaired Student’s *t* test). Data are presented as mean ± SD. *P < 0.05, **P < 0.01, ***P < 0.001. Individual data points in (M) represent single animals, and bars show mean ± SD from 2 independent experiments. Result in (A) to (C), (F) to (H), (J), (K), and (O), individual data points represent single animals and are shown as mean ± SD from one representative of 2 independent experiments with consistent results.

Given the central role of 5-HT in neuroimmune communication and its enrichment in platelet-dense granules [23,24], we investigated whether platelet-released 5-HT mediates AD-related pathology. Enzyme-linked immunosorbent assay (ELISA) analyses revealed that both optogenetic photostimulation and MC903 treatment significantly increased cutaneous 5-HT levels (Fig. 4A and B), an effect reduced by platelet depletion (Fig. 4C), confirming platelets as the predominant 5-HT source in inflamed skin.

To genetically test the requirement for platelet-derived serotonin, we used the tryptophan hydroxylase 1-deficient (*TPH1*^−/−^) mice, which lack peripheral 5-HT (Fig. 4D). At baseline, *TPH1*^−/−^ mice displayed normal skin and behavior (Fig. S20). However, following MC903 treatment, these mice exhibited dramatically reduced ear swelling, inflammatory infiltration, dermal thickening, and spontaneous scratching compared to controls (Fig. 4E to K). In line with these behavioral findings, both macrophage infiltration and C-fiber hyperexcitability were significantly diminished in *TPH1*^−/−^ mice (Fig. 4L to O and Fig. S21). These results indicate that platelet-derived 5-HT is a critical mediator of the full expression of AD-associated neuroimmune pathology.

### Platelet-derived chronic itch and macrophage infiltration through 5-HT–HTR2B/HTR7 axis

To directly validate the role of 5-HT in itch and skin inflammation, 5-HT was administered by intradermal injection. Intradermal injection of 5-HT rapidly elicited scratching behavior and ear edema in mice, whereas no obvious vasodilation was observed (Fig. S22). 5-HT injection daily for 11 consecutive days significantly induced erythema, increased ear thickness, and robust scratching behavior (Fig. S23A to E). Histology following 11 d revealed dense immune cell infiltration and keratinocyte proliferation (Fig. S23F to H). Immunofluorescence analysis demonstrated that 5-HT promoted macrophage infiltration in the ear skin (Fig. S23I and J). Furthermore, Evans blue staining and CD41 immunofluorescence staining showed an increase in vascular permeability and vascular diameter after consecutive injection of 5-HT, which promote platelet infiltration (Fig. S24). These findings indicated that 5-HT could induce itch and macrophage infiltration.

**Fig. 5.**
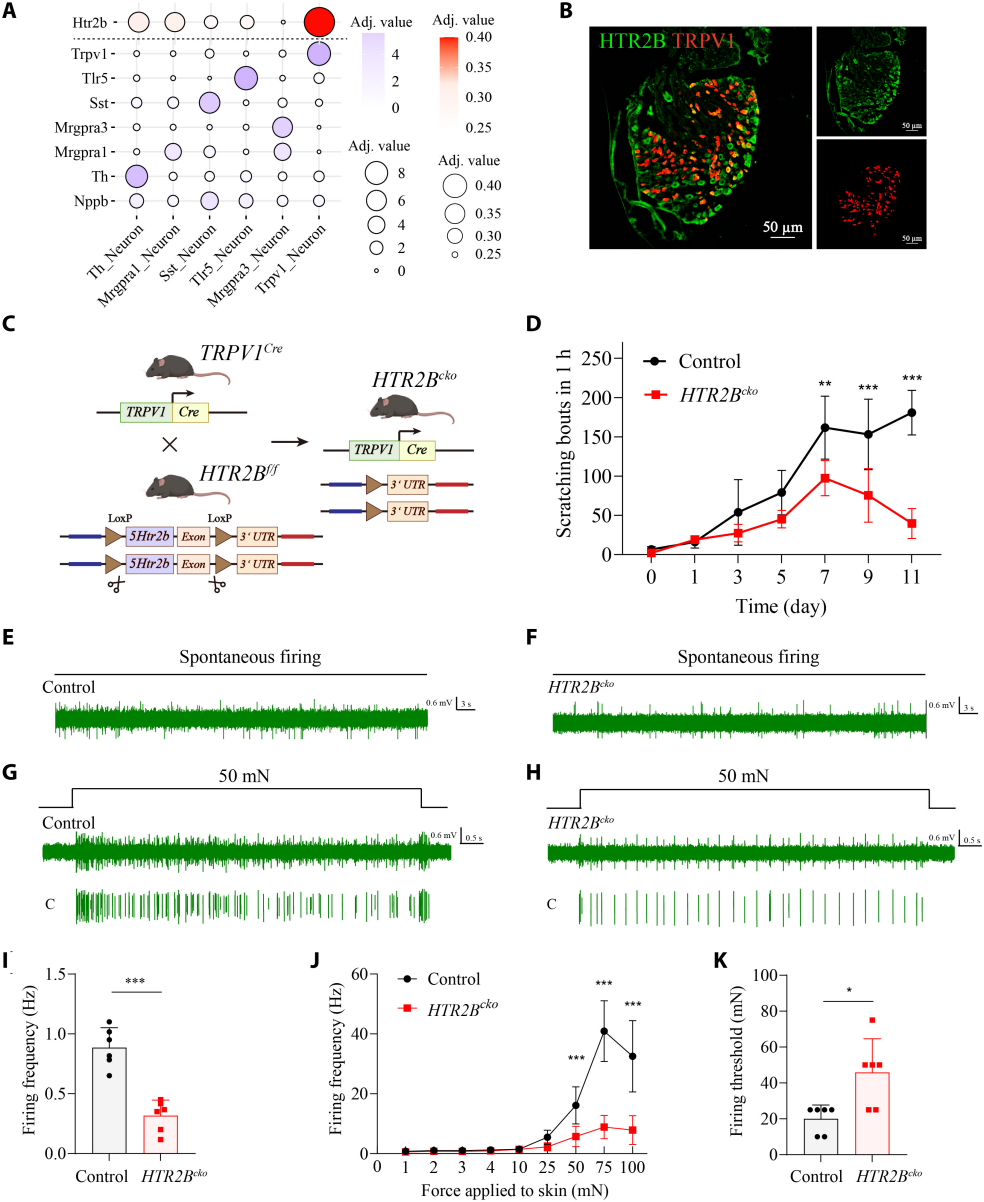
Platelet-derived serotonin activated TRPV1^+^ pruriceptors via HTR2B to promote MC903-induced itch. (A) Bubble plot showed the transcript expression of *Htr2b* in various itch-related neurons based on DRG scRNA-seq data. *n* = 5 mice. (B) Representative images of HTR2B and TRPV1 fluorescence intensity in DRG. *n* = 3 to 5 sections from 3 mice. Scale bar, 50 μm. (C) Schematic diagram of the generation of *TRPV1^Cre^; HTR2B^f/f^* mice (*HTR2B^cko^*). (D) Quantification of spontaneous scratching bouts in *HTR2B^cko^* mice and control littermates. *n* = 5 *HTR2B^cko^* mice, *n* = 6 control littermates. *F*_6,63_ = 9.668, *P* < 0.0001. Day 7: *P* = 0.0012, day 9: *P* < 0.0001, day 11: *P* < 0.0001 (2-way ANOVA followed by Šídák’s multiple comparisons test). (E and F) Representative traces of spontaneous C-fiber firings recorded ex vivo from the control littermates (left) and *HTR2B^cko^* mice (right). (G and H) Representative traces of mechanically evoked C-fiber firings in response to 50-mN force. (I to K) Summarized data showing spontaneous C-fiber firing frequency (I), mechanically evoked C-fiber firing frequency (J), and mechanical firing threshold (K). *n* = 6 units from 4 mice. For (I), *t_10_* = 6.604, *P* < 0.0001 (2-tailed unpaired Student’s *t* test). For (J), *F*_8,90_ = 23.39, *P* < 0.0001. 50 mN: *P* = 0.0007, 75 mN: *P* < 0.0001, 100 mN: *P* < 0.0001 (2-way ANOVA followed by Šídák’s multiple comparisons test). For (K), *P* = 0.0325 (2-tailed Mann–Whitney test). *P < 0.05, **P < 0.01, ***P < 0.001. Individual data points in (I) to (K) represent single animals, and bars show mean ± SD from 2 independent experiments. Result in (D), individual data points represent single animals and are shown as mean ± SD from one representative of 2 independent experiments with consistent results.

Because serotonin can directly activate sensory neurons, we next mapped expression of its receptor HTR2B across dorsal root ganglion (DRG) neurons using single-cell RNA-seq (scRNA-seq). *Htr2b* expression was enriched in subsets of TRPV1^+^ nociceptors (Fig. 5A), and immunostaining confirmed HTR2B protein localization within TRPV1^+^ sensory neurons (Fig. 5B). To test the functional relevance of this pathway, we generated TRPV1-specific HTR2B conditional knockout mice (Fig. 5C). Although *HTR2B^cko^* mice exhibited normal baseline skin structure and behavior (Fig. S25), MC903-treated mutants showed significantly reduced spontaneous scratching (Fig. 5D) and diminished C-fiber excitability (Fig. 5E to K) compared to littermate controls. These findings establish that platelet-derived 5-HT directly activates TRPV1^+^ pruriceptors via HTR2B to drive AD-associated itch.

**Fig. 6.**
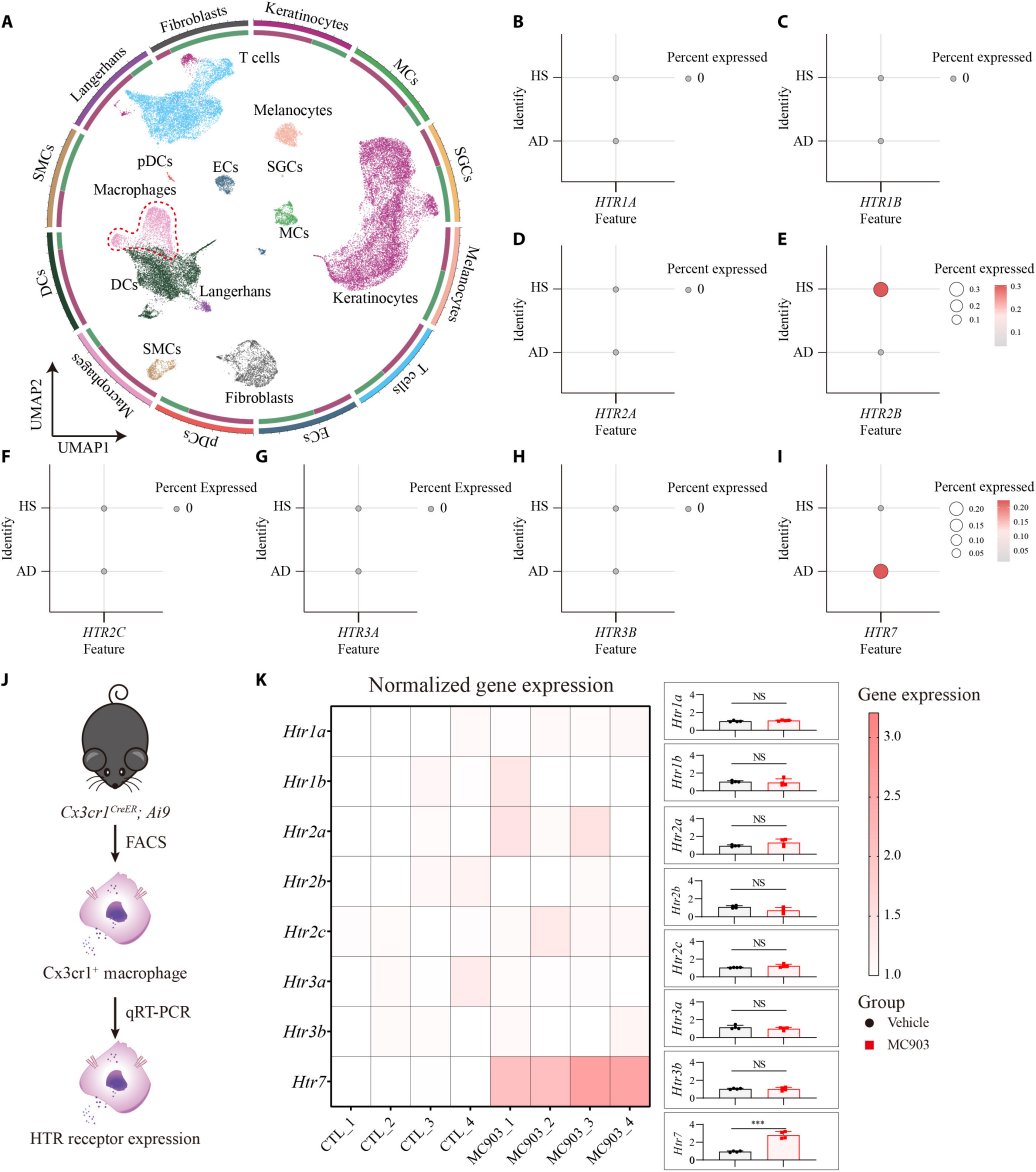
The expression landscape of HTRs in skin macrophages. (A) UMAP visualization and clustering of skin cells from AD patients and healthy individuals (GSE153760, platform: GPL21290; 7 healthy individuals, 8 AD patients). (B to I) Expression patterns of *HTRs* in skin macrophages. (J) Sorting of red fluorescent macrophages by fluorescence-activated cell sorting (FACS). (K) Expression of *Htrs* in Cx3cr1^+^ macrophages from vehicle- or MC903-treated mice. *n* = 4 per group. *t_6_* = 9.256, *P* < 0.0001 (2-tailed unpaired Student’s *t* test). Individual data points in (K) represent single animals and are shown as mean ± SD from one representative of 2 independent experiments with consistent results. MCs, mast cells; SGCs, sweat gland cells; ECs, endothelial cells; pDCs, plasmacytoid dendritic cells; DCs, dendritic cells; SMCs, smooth muscle cells.

**Fig. 7.**
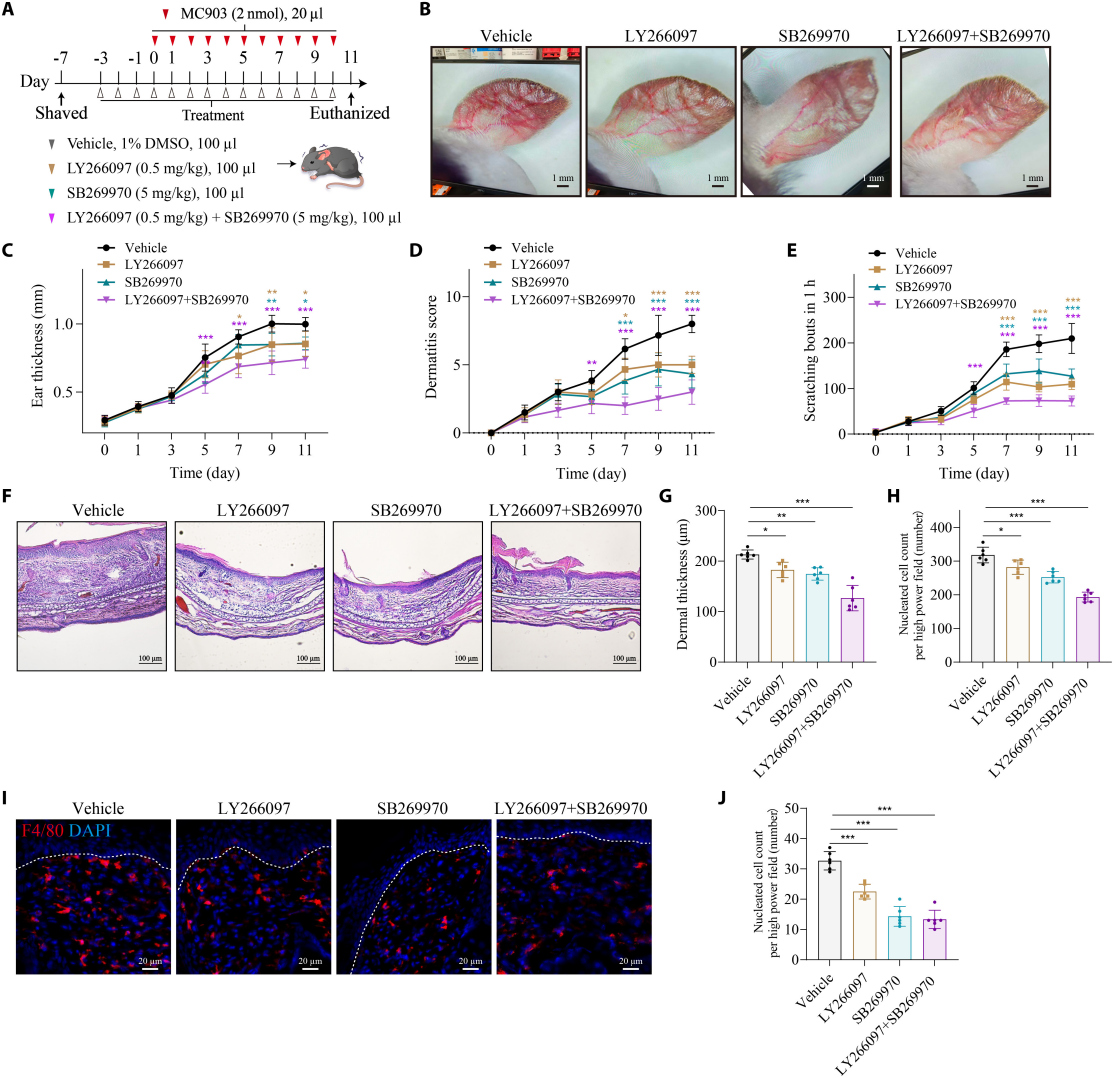
HTR antagonist treatment attenuates pruritus and skin inflammation in MC903-induced AD model. (A) Schematic illustration of HTR2B inhibitor (LY266097) and/or HTR7 inhibitor (SB269970) applications in MC903-induced AD model. (B) Representative images of ears from MC903-induced AD model after 11 d of HTR2B inhibitor and/or HTR7 inhibitor applications. Scale bar, 1 mm. (C and D) Ear thickness (C) and inflammation score (D) measured at the indicated time points. *n* = 6 mice per group. For (C), *F*_18,140_ = 3.900, *P* < 0.0001. Vehicle versus LY266097: day 7: *P* = 0.0174, day 9: *P* = 0.0050, day 11: *P* = 0.0110. Vehicle versus SB269970: day 9: *P* = 0.0050, day 11: *P* = 0.0174. Vehicle versus LY266097 + SB269970: day 5: *P* < 0.0001, day 7: *P* < 0.0001, day 9: *P* < 0.0001, day 11: *P* < 0.0001. For (D), *F*_18,140_ = 8.957, *P* < 0.0001. Vehicle versus LY266097: day 7: *P* = 0.0256, day 9: *P* < 0.0001, day 11: *P* < 0.0001. Vehicle versus SB269970: day 7: *P* < 0.0001, day 9: *P* < 0.0001, day 11: *P* < 0.0001. Vehicle versus LY266097 + SB269970: day 5: *P* = 0.0064, day 7: *P* < 0.0001, day 9: *P* < 0.0001, day 11: *P* < 0.0001 (2-way ANOVA followed by Šídák’s multiple comparisons test). (E) Quantification of spontaneous scratching bouts. *n* = 6 mice per group. *F*_18,140_ = 21.76, *P* < 0.0001. Vehicle versus LY266097: day 7: *P* < 0.0001, day 9: *P* < 0.0001, day 11: *P* < 0.0001. Vehicle versus SB269970: day 7: *P* < 0.0001, day 9: *P* < 0.0001, day 11: *P* < 0.0001. Vehicle versus LY266097 + SB269970: day 5: *P* < 0.0001, day 7: *P* < 0.0001, day 9: *P* < 0.0001, day 11: *P* < 0.0001 (2-way ANOVA followed by Šídák’s multiple comparisons test). (F) Representative H&E histopathology images of ear sections. *n* = 3 to 5 sections from 3 mice. Scale bar, 100 μm. (G and H) Quantification of dermal thickness (G) and dermal immune cell infiltration (H). *n* = 6 mice per group. For (G), *F*_3,20_ = 28.03, *P* < 0.0001. Vehicle versus LY266097: *P* = 0.0032. Vehicle versus SB269970: *P* = 0.0002. Vehicle versus LY266097 + SB269970: *P* = 0.0002. For (H), *F*_3,20_ = 45.05, *P* < 0.0001. Vehicle versus LY266097: *P* = 0.0124. Vehicle versus SB269970: *P* = 0.0018. Vehicle versus LY266097 + SB269970: *P* < 0.0001 (one-way ANOVA followed by Holm–Šídák’s multiple comparisons test). (I and J) Representative images of F4/80^+^ macrophages in the skin, which were quantified in (J). Scale bar, 20 μm. *n* = 6 mice per group. For (J), *F*_3,20_ = 55.62, *P* < 0.0001. Vehicle versus LY266097: *P* < 0.0001. Vehicle versus SB269970: *P* < 0.0001. Vehicle versus LY266097 + SB269970: *P* < 0.0001 (one-way ANOVA followed by Holm–Šídák’s multiple comparisons test). *P < 0.05, **P < 0.01, ***P < 0.001. Individual data points in the remaining panels represent single animals and are shown as mean ± SD from 2 independent experiments.

To further explore the role of 5-HT in macrophage infiltration, we re-examined scRNA transcriptomic data and found the *HTR2B* and *HTR7* expression were enriched in macrophages, while the *HTR7* was significantly increased in AD (Fig. 6A to I). Consistent with the scRNA analysis, our quantitative polymerase chain reaction (qPCR) data further validated the increasing expression of *Htr7* in CX3CR1^+^ skin macrophages from MC903-induced AD mice (Fig. 6J and K). We further assess the therapeutic potential of HTR2B and/or HTR7 antagonist for AD. Of note, HTR2B antagonist, HTR7 antagonist, or combined with these 2 antagonists ameliorated itch and skin inflammation, while the combined treatment produced a more pronounced effect (Fig. 7). Additionally, the application of HTR2B or HTR7, as well as combined HTR2B and HTR7, alleviated itch and skin inflammation in mice, with the combination treatment showing a greater therapeutic effect (Fig. S26).

## Discussion

Our findings identify platelets as essential, previously underappreciated orchestrators of cutaneous neuroimmune regulation. By leveraging optogenetic tools to induce cell-specific activation, we provide evidence supporting a causal role that platelets are sufficient to trigger a cascade of events leading to erythema, edema, and robust pruritic behavior. This study extends emerging paradigms that place platelets at the intersection of the vascular, immune, and neural systems, suggesting that these anucleate fragments do not merely facilitate hemostasis but actively shape the tissue-level inflammatory landscape in AD.

A striking anatomical observation in our study is the robust extravascular accumulation of CD41^+^ platelets in the dermis following activation. In healthy tissue, platelets are predominantly confined to the vascular lumen. However, across multiple models including optogenetic activation, MC903-induced AD, and intradermal 5-HT administration, we observed platelets transmigrating into the interstitial space and closely associating with PGP9.5^+^ sensory nerve endings. This observation raises fundamental questions regarding the mechanisms of platelet extravasation. Our supplementary data using the Evans Blue Miles assays and CD31 whole-mount staining demonstrated significant vascular hyperpermeability and vasodilation following platelet activation. This suggests that activated platelets may release mediators like 5-HT or vascular endothelial growth factor (VEGF), which compromise endothelial integrity, allowing for passive leakage into the dermis [25,26]. Nevertheless, we cannot rule out active transendothelial migration. Platelets express various adhesion molecules, including P-selectin and integrins, which are known to facilitate docking and transmigration across inflamed endothelia [8]. This extravascular presence allows platelets to function as “peripheral neuromodulators”, creating a local microenvironment where high-concentration mediator release can directly influence the firing threshold of sensory fibers.

Beyond passive leakage, platelets actively orchestrate the recruitment of macrophages through the localized establishment of chemokine gradients [27]. Upon activation, platelets degranulate to release α-granule proteins such as CXCL4 and CCL5, which are known to form functional heteromers that synergistically enhance monocyte and macrophage recruitment [8]. Importantly, we identified that platelet-derived 5-HT functions as a parallel regulatory signal for immune infiltration. Our scRNA-seq and qPCR analysis of sorted skin macrophages further revealed a significant enrichment of *Htr2b* and *Htr7*. While HTR2B and HTR7 are traditionally recognized for its role in sensory transmission, its expression on skin-resident macrophages identifies it as a critical mediator of 5-HT-induced chemotaxis in the inflamed skin [28,29]. The therapeutic efficacy of dual HTR2B/HTR7 blockade in reducing macrophage infiltration confirms that 5-HT drives immune recruitment via a multi-receptor signaling network. Furthermore, macrophage-derived signals, such as tumor necrosis factor-α (TNF-α) and interleukin-1β (IL-1β), are documented to further trigger platelet degranulation, suggesting that a potential feedback loop may sustain the chronicity of AD lesions even after the initial trigger is removed [30–32].

Mechanistically, we identified the 5-HT/HTR2B axis on TRPV1^+^ pruriceptors as the primary driver of neurosensitization in our models. Serotonergic itch is predominantly non-histaminergic and resistant to traditional antihistamines. While HTR7 has been established as a mediator of acute and chronic itch via TRPA1 channels [33], our scRNA-seq and lineage-specific genetic ablation data highlight a critical role for HTR2B. This is particularly relevant given that HTR2B activation is linked to pruritus induced by certain antidepressants like sertraline, acting independently of HTR7. HTR2B is a Gq-coupled receptor that likely sensitizes TRPV1 channels via the phospholipase C (PLC) pathway, lowering the firing threshold of C fibers [34]. This is strongly supported by our ex vivo skin–nerve recordings, where *HTR2B^cko^* mice exhibited significantly reduced spontaneous and mechanically evoked firings. This specific receptor–channel coupling explains the transition from acute signaling to chronic peripheral sensitization observed in our models.

From a translational perspective, our study provides a mechanistic rationale for repurposing anti-platelet therapies in dermatology. We demonstrate that clopidogrel, a clinically approved P2Y12 inhibitor, significantly attenuates erythema, ear swelling, and scratching behavior across optogenetic and MC903 models. These findings align with human clinical data reporting elevated systemic platelet activation markers, such as PF4 and β-thromboglobulin, in AD and psoriasis patients, which directly correlate with disease severity and decrease following treatment [6,7]. Given the high safety profile of these drugs in cardiovascular medicine, targeting the platelet–immune–neuron axis represents a promising direction for managing refractory inflammatory skin disorders.

In summary, we propose a framework in which platelets act as a hub within the cutaneous neuroimmune network. By integrating vascular signals and releasing neuromodulatory molecules, platelets bridge the gap between immune recruitment and neural sensitization. Future studies utilizing human skin biopsies and single-cell spatial transcriptomics will be essential to validate these interactions in AD patients and fully explore the therapeutic potential of this platelet–immune–neuron axis.

## Materials and Methods

### Study design

To systematically define the functional role of platelets in cutaneous neuroimmune signaling, we implemented a multidisciplinary experimental strategy. We utilized a *PF4^Cre^; Ai32* optogenetic model to achieve cell type-specific and spatiotemporally precise activation of platelets. This was complemented by pharmacological and genetic platelet depletion strategies in an MC903-induced AD model. To characterize the transcriptomic landscape, we performed transcriptomic profiling of ear tissue and DRG neurons. We further employed flow cytometry to quantify skin-infiltrating immune cell populations. These experimental datasets were integrated with bioinformatic re-analysis of public human AD transcriptomic datasets to identify conserved signaling modules. Mechanistic necessity was interrogated via *Tph1* genetic ablation and neuron-specific deletion of the serotonin receptor *HTR2B*. Finally, we performed pharmacological proof-of-concept studies using a combined regimen of anti-platelet agents and HTR2B/HTR7 antagonists. All experiments included age- and sex-matched littermate controls, with blinding and randomization procedures applied to ensure objective data acquisition and analysis.

### Animals

C57BL/6J mice (#000664), *TRPV1^Cre^* (*TRPV1^tm1(cre)Bbm/J^*, #017769), *CX3CR1^CreER^* (#021160), *iDTR* (#007900), and *Ai32* (#024109) mice were obtained from the Jackson Laboratory (Bar Harbor, ME, USA). *TPH1^−/−^* (#21990), *PF4^Cre^* (#56744), and *HTR2B^fl/fl^* (#15559) mice were purchased from Cyagen Biosciences (Suzhou, China). *Ai9* (#T058417) mice were purchased from GemPharmatech (Nanjing, China). Male and female mice (8 to 12 weeks old) were maintained on a controlled environmental condition (individually ventilated cages, 12-h light/dark cycle and ad libitum food and water). Mice used within each experimental comparison were bred to generate littermates, cohoused under identical conditions, and randomized by investigators blinded to the experimental design. Scratching behavior and dermatitis severity did not differ significantly between males and females. All experiments were ethically approved and conducted in compliance with relevant guidelines of the Institutional Animal Care and Use Committee (IACUC) of the Shanghai Institute of Materia Medica, Chinese Academy of Sciences.

### Sample preparation for bulk RNA-seq and scRNA-seq

For bulk RNA-seq, mice were euthanized on day 11, and ear tissues were collected and snap-frozen in liquid nitrogen. Total RNA was isolated from each sample. Libraries were prepared according to the manufacturer’s instructions and sequenced on the Illumina NovaSeq X Plus platform. Gene expression levels were quantified using RSEM (v1.2.25) and normalized to fragments per kilobase of transcript per million mapped reads (FPKM). Then, raw data underwent background correction, quantile normalization, and probe summarization in the *R* environment for further analysis. Subsequently, principal components analysis (PCA) and unsupervised clustering analysis were utilized to identify the distinct gene expression patterns using the “*prcomp*” function of the *R* package “*stats*”. Immune infiltration analysis was performed using the “*ImmuCellAI*” package. GO enrichment analyses was used to reveal the indeed signaling using the “*clusterProfiler*”, “*enrichplot*”, and “*ggplot2*” packages.

For scRNA-seq, DRG isolation was performed as previously described [35]. Without any treatment, mouse DRGs from T6 to T10 were dissected and immediately placed into ice-cold Hanks’ balanced salt solution (HBSS). The collected DRGs were dissociated in neuron medium [Dulbecco’s modified Eagle’s medium (DMEM)/F12 + 1% penicillin/streptomycin + 12.5 mM glucose] containing 40 U papain, 4 mg/ml collagenase, 10 mg/ml bovine serum albumin (BSA), 1 mg/ml hyaluronidase, and 0.6 mg/ml deoxyribonuclease (DNase) at 37 °C for 30 min. The dissociated neurons were then filtered through a 70-μm cell strainer and washed 3 times with neuron medium. The prepared DRG samples were processed according to the 10x Genomics Cell Ranger pipeline. After background correction, quantile normalization, and probe summarization/elimination, uniform manifold approximation and projection (UMAP) analysis was performed to distinguish DRG neuron subtypes. Differentially expressed gene (DEG) analysis was performed through the FindMarkers function.

### GEO data retrieval and analysis

The RNA-seq data of AD patients were obtained from the Gene Expression Omnibus (GEO) database (https://www.ncbi.nlm.nih.gov/geo/) under the accession ID GSE99802 (platform: GPL570; *n* = 53 healthy individuals, *n* = 59 AD patients). The raw data underwent background correction, quantile normalization, and probe summarization in the *R* environment. DEG analysis was performed using the “*limma*” and “*ggplot2*” packages. GSEA was conducted using the “*gsva*” package, identifying enriched biological pathways relevant to AD pathogenesis. Immune infiltration analysis was performed using the “*Xcell*” package. The spatial transcriptomics-seq data of AD patients were obtained from the GEO database under the accession ID GSE197023 (platform: GPL24676; *n* = 6 healthy individuals, *n* = 7 AD patients). Spatial gene expression analysis was performed by Seurat’s label transfer method as previously described [36]. The scRNA-seq data of AD patients were obtained from the GEO database under the accession ID GSE153760 (platform: GPL21290; *n* = 7 healthy individuals, *n* = 8 AD patients). UMAP was used for 2-dimensional visualization. Cell types were annotated according to canonical marker genes. DEG analysis was used to identify the DEGs in macrophages. The RNA-seq data from AD mouse model were retrieved from GEO (ID: GSE246569, platform: GPL24247; *n* = 6 control littermates, *n* = 8 MC903-induced AD mouse model). DEG analysis was performed using the “*limma*” and “*ggplot2*” packages. GSEA was conducted using the “*gsva*” package. Immune infiltration analysis was performed using the “*ImmuCellAI*” package for mouse.

### Optogenetics

Optogenetic stimulation of the mouse dorsal ear was performed using a 473-nm diode-pumped solid-state laser (100 mW output, Laser and Optics Century, Shanghai, China) [37]. An optical fiber cannula was fixed 1 cm above the ear skin surface, illuminating a 1 cm × 1 cm area for stimulation, delivering light at 10 Hz, 30 min per session, with a power density of 8 mW/mm^2^. This protocol was repeated daily for 11 consecutive days. Both *PF4^Cre^; Ai32* transgenic mice and *Ai32* littermate controls were subjected to the same photostimulation regimen.

### MC903-induced AD mouse model

AD-like conditions were induced by topical application of 2 nmol MC903 (calcipotriol, Sigma-Aldrich, dissolved in 20 μl of ethanol) to the dorsal ear once daily for 11 consecutive days. Control mice were treated with ethanol alone. Disease progression was monitored daily by measuring ear thickness with a dial thickness gauge and by scoring dermatitis severity for erythema/hemorrhage, scaling/dryness, excoriation, and edema according to established criteria [38]. All animals were euthanized on day 11, and ear skin was harvested for further evaluation.

### Conditional platelet depletion

To induce platelet ablation, *PF4^Cre^; iDTR* mice were intraperitoneally injected with DTX (20 μg/kg; Sigma) or an equal volume of phosphate-buffered saline (PBS) every other day for a total of 3 doses (days 1, 3, and 5) before the initiation of the experimental procedures. Formal experiments were started after completion of the initial depletion regimen (day 7). To maintain platelet depletion throughout the course of the study, mice continued to receive DTX or PBS every other day during the subsequent 11-d experimental period (days 7 to 17). The efficiency of platelet depletion was verified by platelet counts measured in venous blood samples on days 0, 5, and 17, and only mice with platelet counts reduced to <15% of their individual pre-DTX baseline were included in subsequent analyses.

### Drug administration

To evaluate the therapeutic effect of antiplatelet treatment, mice were administered clopidogrel (HY-15283, 10 mg/kg, MCE, NJ, USA) by oral gavage once daily in a total volume of 100 μl per mouse. Clopidogrel treatment was initiated on day 4 after model induction by 473-nm photostimulation or topical MC903 application and was maintained daily for the duration of the experiment [39]. Vehicle-treated control mice received the same volume of solvent on an identical schedule. Clopidogrel efficacy was verified by prolongation of tail bleeding time in a mouse tail-vein bleeding assay.

To evaluate the therapeutic effect of HTR2B and/or HTR7 inhibition, mice were randomly assigned to receive vehicle [1% dimethyl sulfoxide (DMSO) in PBS], HTR2B inhibitor (LY266097, 0.5 mg/kg, MCE, NJ, USA), HTR7 inhibitor (SB269970, 5 mg/kg, MCE, NJ, USA), or a combination of HTR2B and HTR7 inhibitors. All compounds were administered by intraperitoneal injection once daily in a total volume of 100 μl. Mice were pretreated for 3 consecutive days before the start of the formal experiments, and treatment was continued daily after experimental initiation to maintain pharmacological inhibition throughout the study. Control animals received the corresponding vehicle on the same schedule.

To assess the pruritogenic and pro-inflammatory effects of 5-HT in vivo, mice were subjected to acute or repeated intradermal 5-HT (HY-B1473, 100 μM, 20 μl, MCE, NJ, USA) administration in the ear pinna [33]. For acute challenge, 5-HT was intradermally injected, and scratching behavior was recorded immediately for 30 min. Evans blue extravasation was assessed 30 min after injection to evaluate vascular permeability, as described below. For repeated challenge, mice received daily intradermal injections of 5-HT (100 μM, 20 μl) for 11 consecutive days. Ear thickness and scratching behavior were assessed at the indicated time points during the course of treatment.

### Itch behavior assessment

Itch behavior was assessed using a standardized video-recording protocol [40]. Prior to the assessment of spontaneous itch behavior, mice were acclimated to the testing chambers for at least 3 days before the experiment. All recordings were conducted in a dedicated behavioral suite under controlled lighting conditions and in isolation from external noise. Cameras were positioned at an elevated 45° angle to ensure unobstructed capture of full-body movements at a recording rate of 30 frames per second. On the experimental day, mice were filmed individually for 60 min. Recorded videos were scored independently by 2 researchers who were blinded to genotype and experimental groups. To assess inter-rater reliability, a randomly selected subset of 30% of the recordings was independently scored by both raters using the same predefined criteria, and agreement was quantified using the intraclass correlation coefficient (ICC > 0.75, 2-way random-effects model, absolute agreement). A scratching bout was defined as one or more rapid, repetitive strokes of the hind paw directed to the MC903-treated dorsal ear, ending when the paw was placed back on the floor or when licking/biting of the toes occurred.

### Open-field test

Open-field behavior was assessed using an automated open-field system (Xinruan Information Technology Co. Ltd., Shanghai, China). The apparatus consisted of a square arena placed within a sound-attenuating cubicle. Mice were individually placed in the chamber for a single 10-min session, during which total distance traveled, rearing behavior, and habituation rate were analyzed by the system’s software. After each trial, the apparatus was cleaned at least 3 times with ethanol.

### Evans blue assay for vascular leakage

To assess vascular leakage in the mouse ear, Evans blue was dissolved in sterile PBS at 20 mg/ml and administered intravenously at 100 μl per 20 g of body weight. Thirty minutes later, ear tissues were collected for analysis. For quantification, the tissues were incubated in formamide at 60 °C overnight to extract Evans blue, and absorbance was measured at 620 nm. Evans blue content was normalized to tissue weight to quantify vascular extravasation. Representative images were acquired under identical conditions for visualization.

### Hematoxylin and eosin staining assay

Whole ear tissues were harvested, fixed in 4% paraformaldehyde (PFA), embedded in optimal cutting temperature (OCT) with a consistent cross-sectional orientation, and serially sectioned at 12 μm. Hematoxylin and eosin (H&E) staining was performed on the prepared 12-μm mouse ear sections. The steps are as follows: hematoxylin (1 min), washed with PBS (5 min × 3), 1% acid alcohol differentiation (5 s), eosin (2 min), washed with PBS (5 min × 3). Tissues were dehydrated, cleared, and mounted with neutral gum and then visualized with a bright-field microscope. In photostimulated or AD samples, section selection was centered on the photostimulated or lesion-related region; in control samples, the corresponding area at the same relative anatomical level was used for comparison. Representative H&E images were chosen only from sections showing comparable orientation and tissue architecture across groups. Multiple serial sections from each ear were reviewed, and only sections with comparable anatomical level and orientation were used for representative imaging.

### Whole-mount immunofluorescence staining of mouse ears

Whole-mount staining was performed as previously described [41,42]. Mouse ears were harvested and fixed in 4% PFA for 24 h, followed by extensive washing with PBS. After 3 washes in PBS (10 min each), the tissues were incubated with primary antibodies diluted in PBS containing 10% donkey serum and 20% DMSO at room temperature (RT) for 5 d (rat anti-CD31, 1:100; BD Biosciences, 550274). The samples were then washed with PBS every 10 min for 2 h and incubated with Alexa Fluor 647-conjugated secondary antibodies in PBS containing 10% donkey serum and 20% DMSO at RT for 3 d. After a final series of washes in PBS every 10 min for 2 h, the samples were imaged using a confocal microscope. Three-dimensional reconstruction was performed using Leica Application Suite X (LAS X), and images were processed in ImageJ or Adobe Photoshop for publication.

### Immunofluorescence assay

Collagen-coated slides were prepared by incubation with collagen solution (#SLCL8775, 0.05 mg/ml; Sigma, St. Louis, MO, USA) before sample collection. Fresh mouse whole blood was directly applied to the coated slides and immediately spread to generate blood smears. After air drying, the smears were fixed in 4% PFA for 10 min. The prepared 12-μm mouse ear sections or blood smears were blocked with 10% normal donkey serum for 2 h at RT. The samples were incubated overnight at 4 °C with primary antibodies (rabbit anti-PGP9.5, 1:500, Abcam, ab108986; mouse anti-CD41, 1:300, BD Pharmingen, #553847; rat anti-F4/80, 1:100, Santa Cruz, sc-52664; rabbit anti-HTR2B, 1:300, Sigma, #58512). The following day, the sections were washed with PBS and incubated for 2 h at RT with species-appropriate Alexa Fluor-conjugated secondary antibodies (Thermo Fisher Scientific). After additional washes, nuclei were counterstained with 4′,6-diamidino-2-phenylindole (DAPI) (1 μg/ml) for 5 min.

### Ex vivo skin–nerve preparations

Ex vivo skin–nerve recordings were performed on mouse skin samples as previously described [43–45]. A segment of skin was dissected and cleared of underlying tissue. The corresponding nerve bundle was isolated into an adjacent oil-filled chamber and mounted on a gold recording electrode. Spontaneous C-fiber activity was recorded for 30 min. Mechanically evoked C-fiber firings were measured through a dual-mode lever system (Aurora Scientific Inc.) delivering ascending forces (1 to 150 mN). Neuronal responses were captured and analyzed using *Spike2* software (Cambridge Electronic Design).

### Enzyme-linked immunosorbent assay

5-HT (serotonin) concentrations were detected using a competitive ELISA kit (Enzyme-linked Biotechnology, Shanghai, China). A 40-mg skin sample was lysed in 300 μl of ice-cold radioimmunoprecipitation assay (RIPA) buffer and homogenized at 4 °C (65 Hz, 3 min). After centrifugation, 50 μl of supernatant was added to a 96-well microplate and incubated with 50 μl of primary antibodies at 37 °C for 30 min. After washing, the samples were incubated with secondary antibodies at 37 °C for 30 min. Finally, the absorbance was determined using a microplate spectrophotometer at 450 nm, and the sample concentration was calculated using a standard curve.

### Flow cytometry analysis and cell sorting

Mouse peripheral blood was collected into sodium citrate-containing tubes and processed immediately to minimize ex vivo platelet activation. For flow cytometric staining, whole blood samples were incubated with fluorophore-conjugated antibodies against peridinin–chlorophyll–protein complex–cyanine 5.5 (PerCP-Cy5.5) anti-mouse CD41 (BD Pharmingen, #757754), phycoerythrin–cyanine 7 (PE-Cy7) anti-mouse CD61 (BD Pharmingen, #755845), and BV605 anti-mouse CD62P (BD Pharmingen, #740358) at a dilution of 1:100 for 20 to 30 min at RT in the dark. After staining, samples were diluted in staining buffer and subjected to flow cytometric analysis.

### Fluorescence-activated cell sorting of CX3CR1-positive skin macrophages

To induce robust Cre activity in the *CX3CR1^CreER^* mouse lines, tamoxifen (100 mg/kg, Sigma, St. Louis, MO, USA) was administered via intraperitoneal injection for 5 consecutive days. Tamoxifen was dissolved in corn oil and freshly prepared daily. All in vivo and in vitro experiments were performed between 7 and 15 d after the final tamoxifen injection. For the preparation of single-cell suspensions from homeostatic back skin, subcutaneous fat was removed with a scalpel. Skin samples were placed dermis-side down on Whatman filter paper and incubated in 0.25% trypsin (Gibco) containing 0.1 mg/ml DNase II (Sigma-Aldrich) at 37 °C for 45 min with gentle shaking to separate the epidermis from the dermis. The epidermis was further digested in 0.25% trypsin for 15 min, whereas the dermis was incubated in RPMI 1640 containing 250 μg/ml Liberase TL (Roche) for 60 to 90 min at 37 °C with gentle shaking. The digested tissues were filtered through a 40-μm cell strainer to remove debris, and the resulting cells were washed and resuspended in fluorescence-activated cell sorting (FACS) buffer. CX3CR1^+^ skin macrophages labeled with red fluorescence were sorted by flow cytometry according to the endogenous fluorescent signal. Sorted cells were collected for downstream RNA isolation and quantitative real-time PCR (qRT-PCR) analysis.

### Quantitative real-time PCR

Total RNA was isolated from sorted Cx3cr1^+^ macrophages using TRIzol Reagent (Invitrogen) according to the manufacturer’s instructions. RNA quantity and quality were determined using a NanoDrop spectrophotometer (Thermo Fisher Scientific). To facilitate RNA precipitation, 12 μl of linear acrylamide was added to each sample. cDNA was synthesized using the Maxima H Minus First Strand cDNA Synthesis Kit (Thermo Fisher Scientific) following the manufacturer’s protocol. Quantitative PCR was performed using Power SYBR Green PCR Master Mix (Bio-Rad). Relative gene expression was calculated by the 2^−ΔΔCt^ method using Gapdh as the internal reference and was normalized to the control group. Primer sequences are listed in Table S1.

### Quantification and statistical analyses

Statistical analyses were performed using GraphPad Prism (GraphPad Software 9.1, San Diego, CA). All quantitative data are expressed as the mean ± standard deviation (SD). Normal distribution was first tested using the Kolmogorov–Smirnov test or Shapiro–Wilk test before further analysis. All data are derived from biological replicates (individual animals or independent cell preparations), and sample sizes (*n*) indicate the number of biological replicates unless otherwise stated. Comparisons between 2 independent groups were performed using an unpaired Student’s *t* test for normally distributed data with equal variances, and the corresponding test statistics were reported in the figure legends as *t_df_* and *P* values. The Mann–Whitney *U* test was used for non-normally distributed data. For experiments involving 3 or more experimental groups, 1-way or 2-way analysis of variance (ANOVA) followed by Šídák’s multiple comparisons test was applied. For 2-way ANOVA, main effects and interaction effects between the 2 factors were assessed, and interaction terms were reported as *F_DFn,DFd_* with corresponding *P* values. Two-tailed correlation analyses were used to assess associations between 2 variables. A priori significance was accepted at *P* < 0.05. Asterisks in the figures denote *P < 0.05, *^*P* < 0.01, and ***P < 0.001; NS indicates no statistically significant difference.

## Data Availability

All data needed to evaluate the conclusions in the paper are present in the paper and/or the Supplementary Materials. Our RNA-sequencing data were available in GEO database (ID: GSE314731). Our scRNA-sequencing data have been deposited in the NCBI Sequence Read Archive (SRA), with accession code PRJNA1456602.
