# Supplementary material for: Platelets Orchestrate a Neuroimmune Axis Driving Cutaneous Inflammation and Itch
Source: Research (Wash D C). 2026 May 28;9:1294. doi: 10.34133/research.1294 (PMC13216866; doi:10.34133/research.1294)
Supplement: Supplementary 1 — Figs. S1 to S26 Table S1 [file research.1294.f1.docx]

Supplementary Materials for

**Platelets Orchestrate a Neuroimmune Axis Driving Cutaneous Inflammation and Itch**

Ximin Hu *et al.*

*Corresponding author. Email: fengjing@simm.ac.cn; eyyangronghua@scut.edu.cn; shaofpu@sjtu.edu.cn

**This PDF file includes:** Figs. S1 to S26 and Table S1


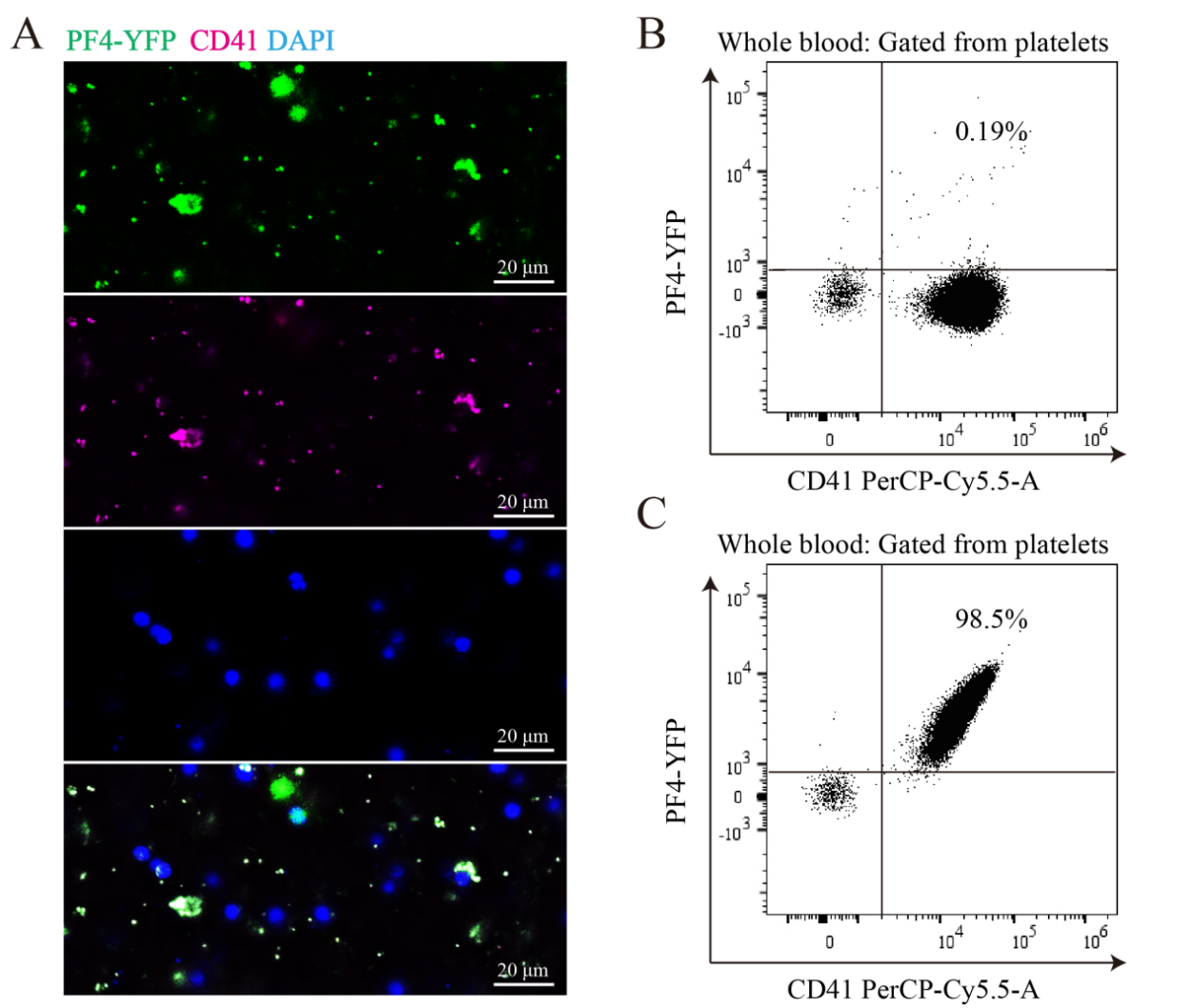


**Fig. S1. Validation of platelet-specific YFP labeling in *PF4^Cre^; Ai32* mice.** (A) Immunofluorescence staining was performed on whole blood from PF4-YFP mice. CD41 was used as a platelet marker, and YFP represented PF4-driven reporter expression. Nuclei were counterstained with DAPI. n = 3-5 sections from 3 mice. Scale bar, 20 μm. (B) In Cre-negative littermate controls, CD41^+^ platelets showed minimal YFP fluorescence. (C) In *PF4^Cre^; Ai32* mice, the majority of CD41^+^ events were YFP^+^, indicating efficient reporter activation in the platelet lineage.


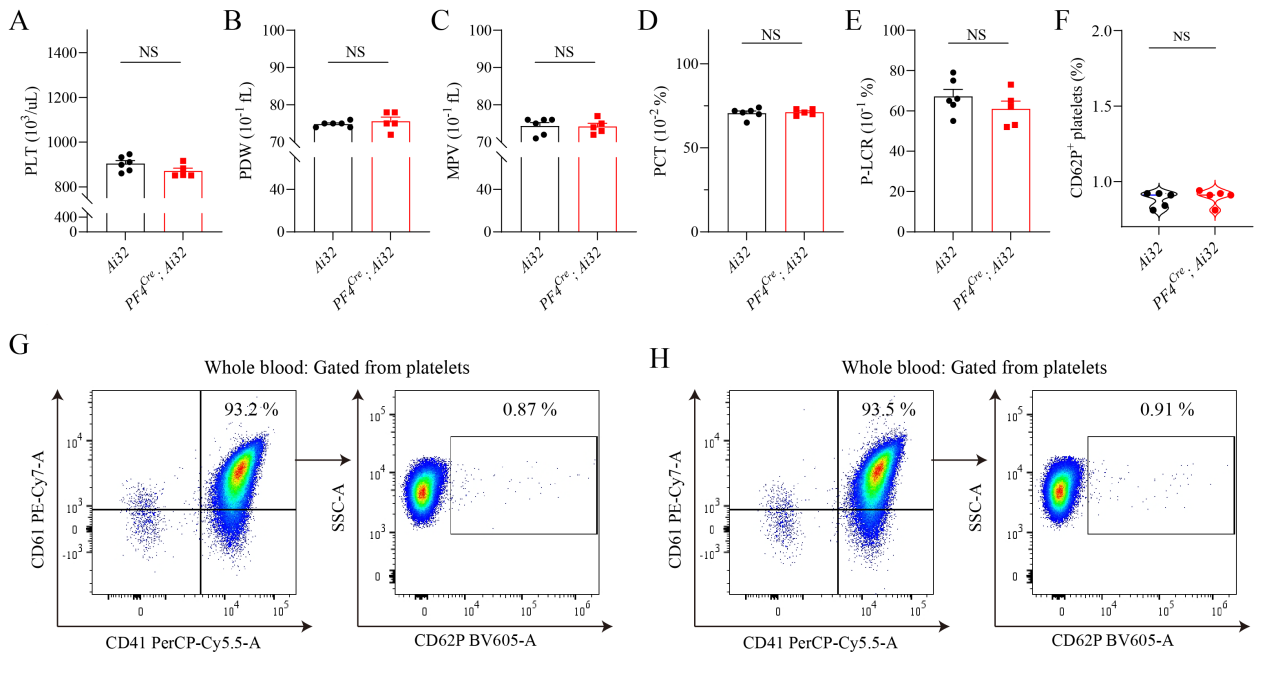


**Fig. S2**. **Hematological and flow cytometric characterization of platelets in *Ai32* and *PF4^Cre^; Ai32* mice under physiological conditions.** (A-E) Changes in platelet parameters (PLT, PCT, MPV, PDW, and P-LCR) in whole blood in *Ai32* and *PF4^Cre^; Ai32* mice under physiological conditions. n = 6 *Ai32* mice and n = 5 *PF4^Cre^; Ai32* mice (two-tailed unpaired Student’s *t* test). (F) Quantification of CD62P expression on CD41^+^/CD61^+^ platelets in *Ai32* and *PF4^Cre^; Ai32* mice under physiological conditions. n = 5 mice per group (two-tailed unpaired Student’s *t* test). (G) Representative expression patterns of CD62P on CD41^+^/CD61^+^ platelets in *Ai32* mice under physiological conditions. (H) Representative patterns of expression of CD62P on CD41^+^/CD61^+^ platelets in *PF4^Cre^; Ai32* mice under physiological conditions. NS, not significant. Results in (A-E) are represented single animals and shown as mean ± SD from one representative of two independent experiments with consistent results. Individual data points in (F) represent single animals from two independent experiments.


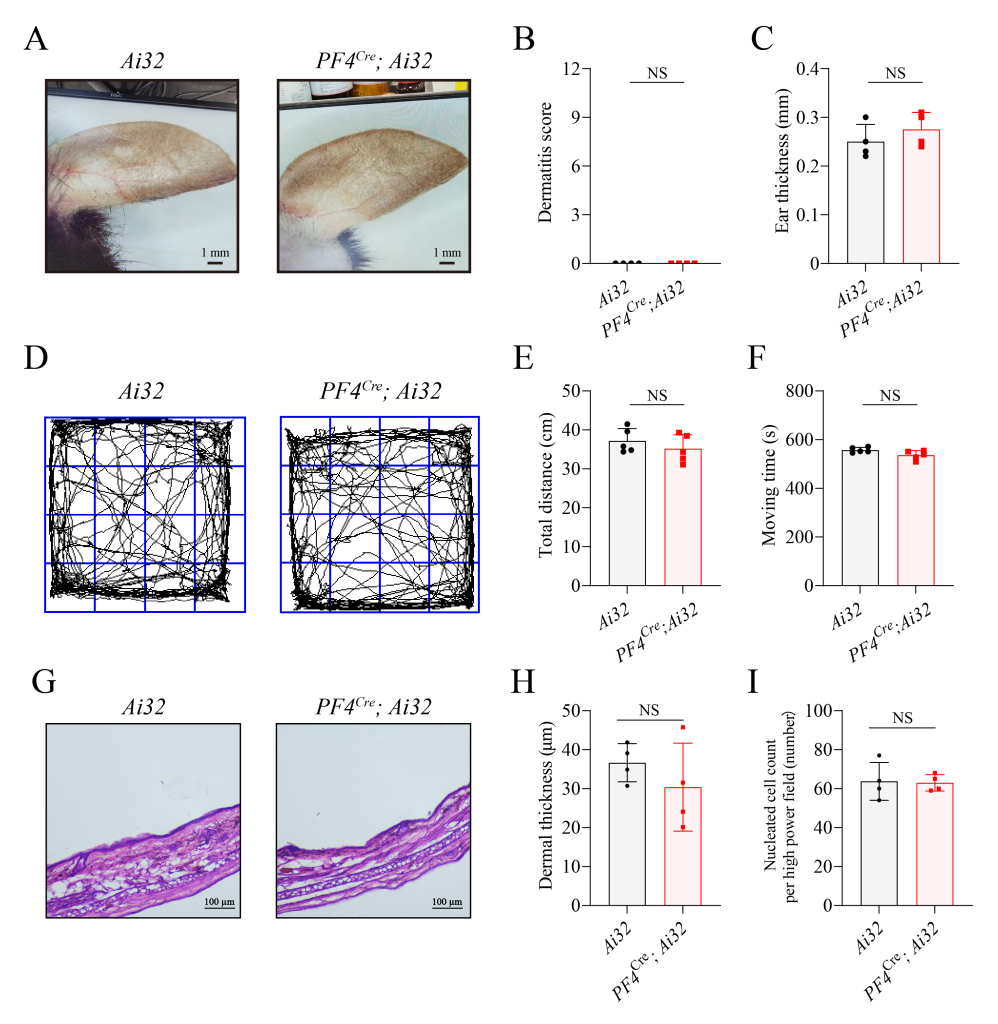


**Fig. S3**. ***PF4^Cre^; Ai32* mice exhibit normal baseline skin morphology or spontaneous behavior.** (A) Representative clinical images of ears from *Ai32* control littermates and *PF4^Cre^; Ai32* mice under basal conditions. Scale bar, 1 mm. (B-C) Quantification of skin dermatitis scores (B) and ear thickness (C). n = 4 mice per group (two-tailed unpaired Student’s *t* test). (D) Representative open-field test results from *Ai32* control littermates and *PF4^Cre^; Ai32* mice under basal conditions. (E-F) Quantification of total distance (E) and moving time (F). n = 5 mice per group (two-tailed unpaired Student’s *t* test). (G) Representative H&E-stained skin sections from *Ai32* control littermates and *PF4^Cre^; Ai32* mice under basal conditions. n = 3-5 sections from 3 mice. (H-I) Quantification of epidermal thickness (H) and dermal immune cell infiltration (I). n = 4 mice per group (two-tailed unpaired Student’s *t* test). NS, not significant. Individual data points in the quantitative panels represent single animals and are shown as mean ± SD from one representative of two independent experiments with consistent results.


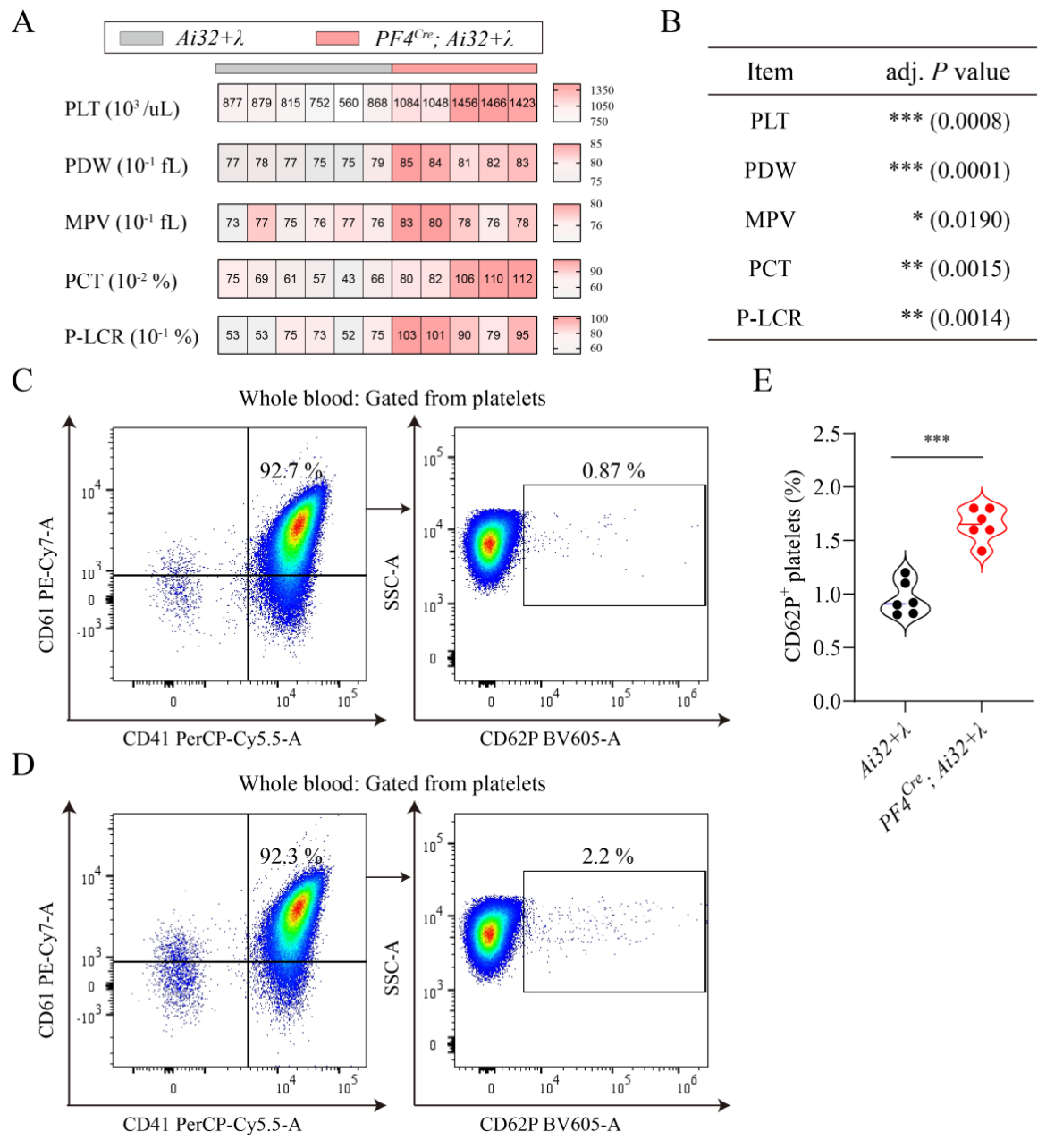


**Fig. S4**. **Optogenetic photostimulation enables controlled activation of platelets in *PF4^Cre^; Ai32* mice**. (A-B) Changes in platelet parameters (PLT, PCT, MPV, PDW, and P-LCR) in whole blood after photostimulation (+λ), as determined by routine blood tests, which were quantified in (B). n = 6 control littermates and n = 5 photostimulated *PF4^Cre^; Ai32* mice. For PLT, *t_9_* = 4.954, *P* = 0.0008 (two-tailed unpaired Student’s *t* test). For PDW, *t_9_* = 6.394 (two-tailed unpaired Student’s *t* test), *P* = 0.0001. For MPV, *t_9_* = 2.853, *P* = 0.0190 (two-tailed unpaired Student’s *t* test). For PCT, *t_9_* = 4.473, *P* = 0.0015 (two-tailed unpaired Student’s *t* test). For P-LCR, *P* = 0.0014 (two-tailed Mann-Whitney test). (C) Representative expression patterns of CD62P on CD41^+^/CD61^+^ platelets in photostimulated *Ai32* mice. (D) Representative expression patterns of CD62P on CD41^+^/CD61^+^ platelets in photostimulated *PF4^Cre^; Ai32* mice. (E) Quantification of CD62P expression on CD41^+^/CD61^+^ platelets in photostimulated *Ai32* and *PF4^Cre^; Ai32* mice. n = 6 mice per group. *t_10_* = 7.741, *P* < 0.0001 (two-tailed unpaired Student’s *t* test). **P* < 0.05, ***P* < 0.01, ****P* < 0.001. Results in (A) and (B) are represented single animals and shown as mean ± SD from one representative of three independent experiments with consistent results. Individual data points in (E) represent single animals from two independent experiments.


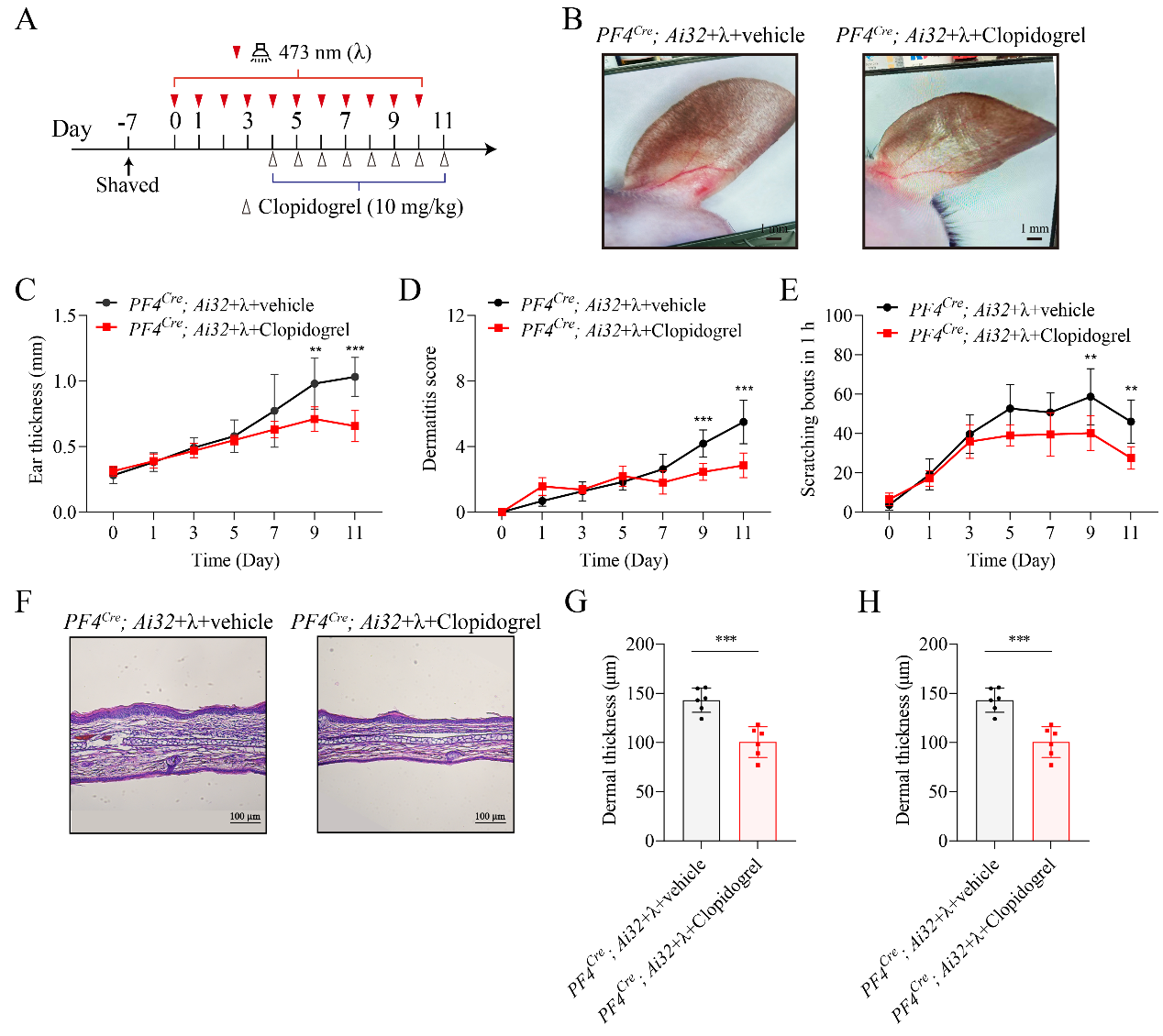


**Fig. S5. Clopidogrel suppressed photostimulation induced cutaneous inflammation and pruritus.** (A) Schematic of the clopidogrel treatment in photostimulated *PF4^Cre^; Ai32* mice. (B) Representative images of ears. Scale bar, 1 mm. (C-D) Ear thickness (C) and inflammation score (D) measured at the indicated time points. n = 6 mice per group. For (C), *F_6,70_* = 4.999, *P* = 0.0003. day 9: *P* = 0.0016, day 11: *P* < 0.0001. For (D), *F_6,70_* = 12.06, *P* < 0.0001. day 9: *P* = 0.0003, day 11: *P* < 0.0001 (two-way ANOVA followed by Šídák’s multiple comparisons test). (E) Quantification of spontaneous scratching bouts. n = 6 mice per group. *F_6,70_* = 2.700, *P* = 0.0204. day 9: *P* = 0.0038, day 11: *P* = 0.0038 (two-way ANOVA followed by Šídák’s multiple comparisons test). (F) Representative H&E histopathology images of ears. n = 3-5 sections from 3 mice. Scale bar, 100 μm. (G-H) Quantification of dermal thickness (G) and dermal immune cell infiltration (H). n = 6 mice per group. For (G), *t_10_* = 5.280, *P* = 0.0004. For (H), *t_10_* = 9.641, *P* < 0.0001 (two-tailed unpaired Student’s *t* test). **P* < 0.05, ***P* < 0.01, ****P* < 0.001. Individual data points in the quantitative panels represent single animals and are shown as mean ± SD from two independent experiments.


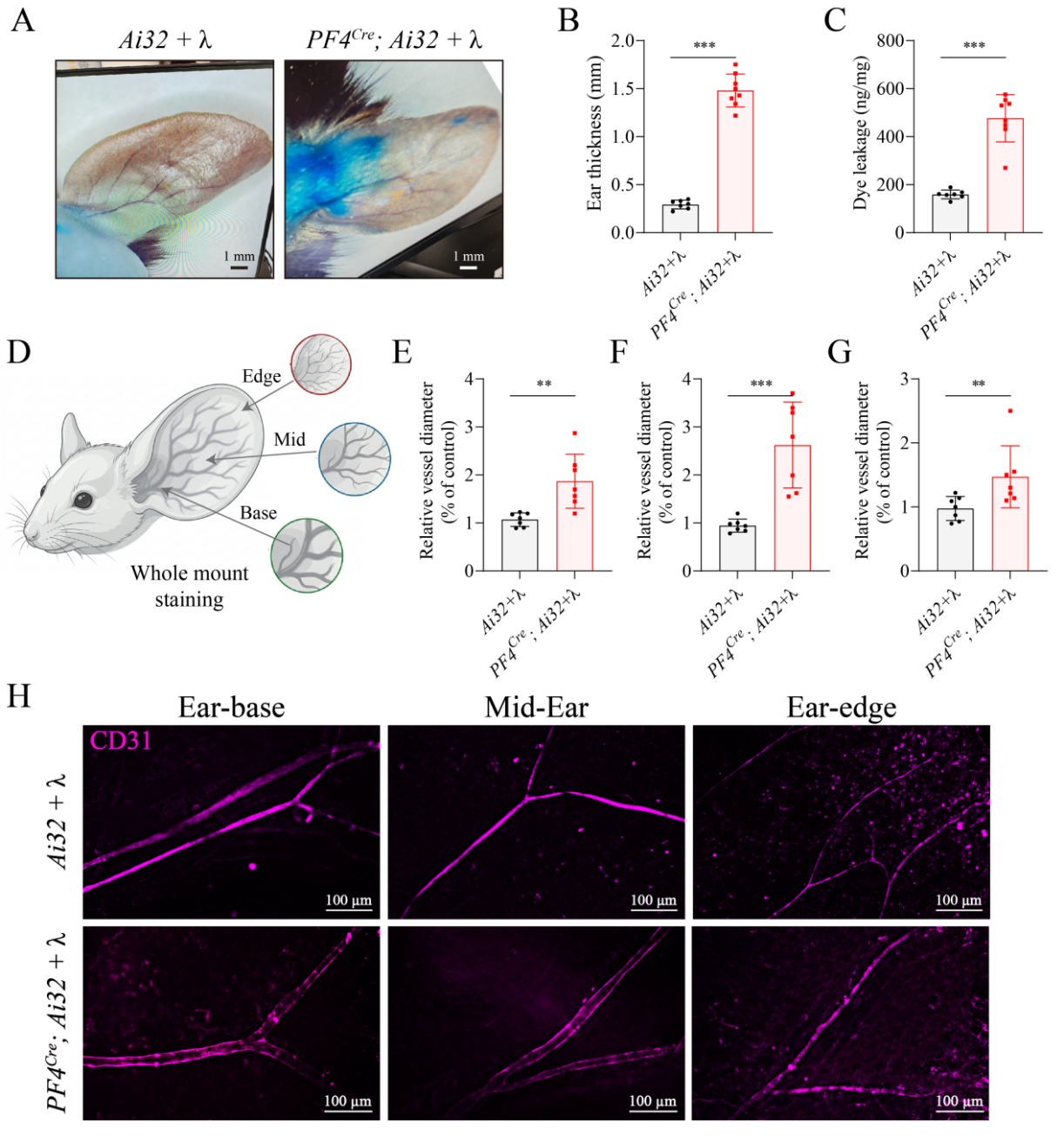


**Fig. S6. Validation of vascular leakage and vasodilation in the ears of the optogenetically photostimulated *PF4^Cre^; Ai32* mice.** (A) Representative photos of Evans blue staining in the ears of photostimulated *Ai32* and *PF4^Cre^; Ai32* mice. (B) Quantification of ear thickness in photostimulated *Ai32* and *PF4^Cre^; Ai32* mice. n = 7 control littermates and n = 8 photostimulated *PF4^Cre^; Ai32* mice. *t_13_* = 17.67, *P* < 0.0001 (two-tailed unpaired Student’s *t* test). (B) Quantification of Evans blue leakage in photostimulated *Ai32* and *PF4^Cre^; Ai32* mice. n = 7 in control littermates and n = 8 photostimulated *PF4^Cre^; Ai32* mice. *t_13_* = 8.362, *P* < 0.0001 (two-tailed unpaired Student’s *t* test). (D) Illustration of the CD31 whole mount staining in mouse ear. (E-G) Quantification of the vascular diameter at the ear base (E), middle (F) and edge (G) based on CD31 whole mount staining. n = 7 mice per group. For (E), *t_12_* = 3.625, *P* = 0.0035 (two-tailed unpaired Student’s *t* test). For (F), *t_12_* = 4.902, *P* = 0.0004 (two-tailed unpaired Student’s *t* test). For (G), *P* = 0.0082 (two-tailed Mann-Whitney test). (H) Representative images of CD31^+^ vascular in the ear of photostimulated *Ai32* and *PF4^Cre^; Ai32* mice. Scale bar, 100 μm. ***P* < 0.01, ****P* < 0.001. Individual data points in the quantitative panels represent single animals and are shown as mean ± SD from two independent experiments.


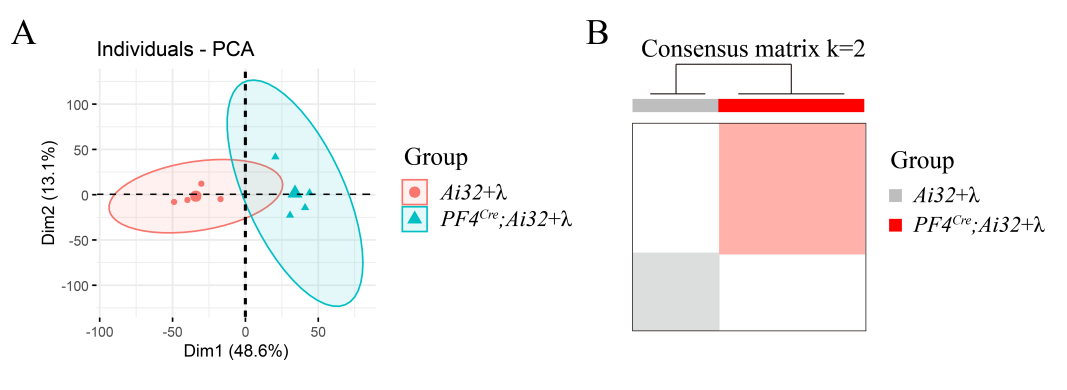


**Fig. S7**. **RNA sequencing analysis of ears from photostimulated *PF4^Cre^; Ai32* mice and the control littermates.** (A-B) PCA analysis (A) and unsupervised clustering analysis (B) of gene landscape between photostimulated *PF4^Cre^; Ai32* mice and control littermates. n = 4 photostimulated *PF4^Cre^; Ai32* mice, n = 4 control littermates.


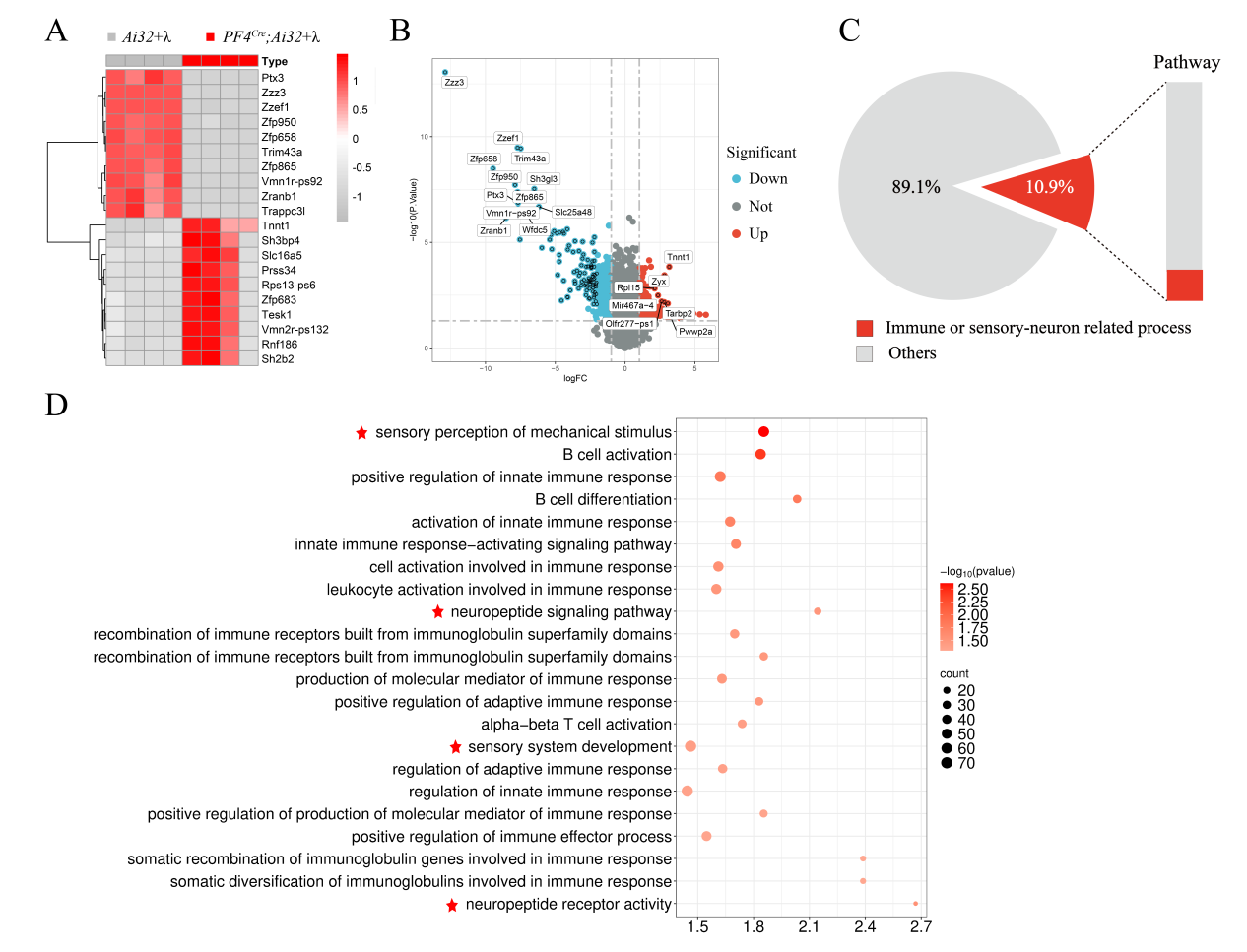


**Fig. S8**. **Immune- and sensory neuron–related pathways are enriched following photostimulation.** (A-B) Heatmap (A) and volcano plot (B) of differentially expressed genes (DEGs). DEGs are defined as adj. *P* < 0.05 and |log_2_FC| > 1. (C) Pie chart displaying the proportion of immune and sensory-neuron related signaling pathways in significantly enriched pathways in photostimulated *PF4^Cre^; Ai32* mice. (D) Bubble plot showed an overview of immune or sensory-neuron related processes based on gene ontology (GO) analysis. Red stars point the sensory-neuron related process. n = 4 photostimulated *PF4^Cre^; Ai32* mice, n = 4 control littermates.


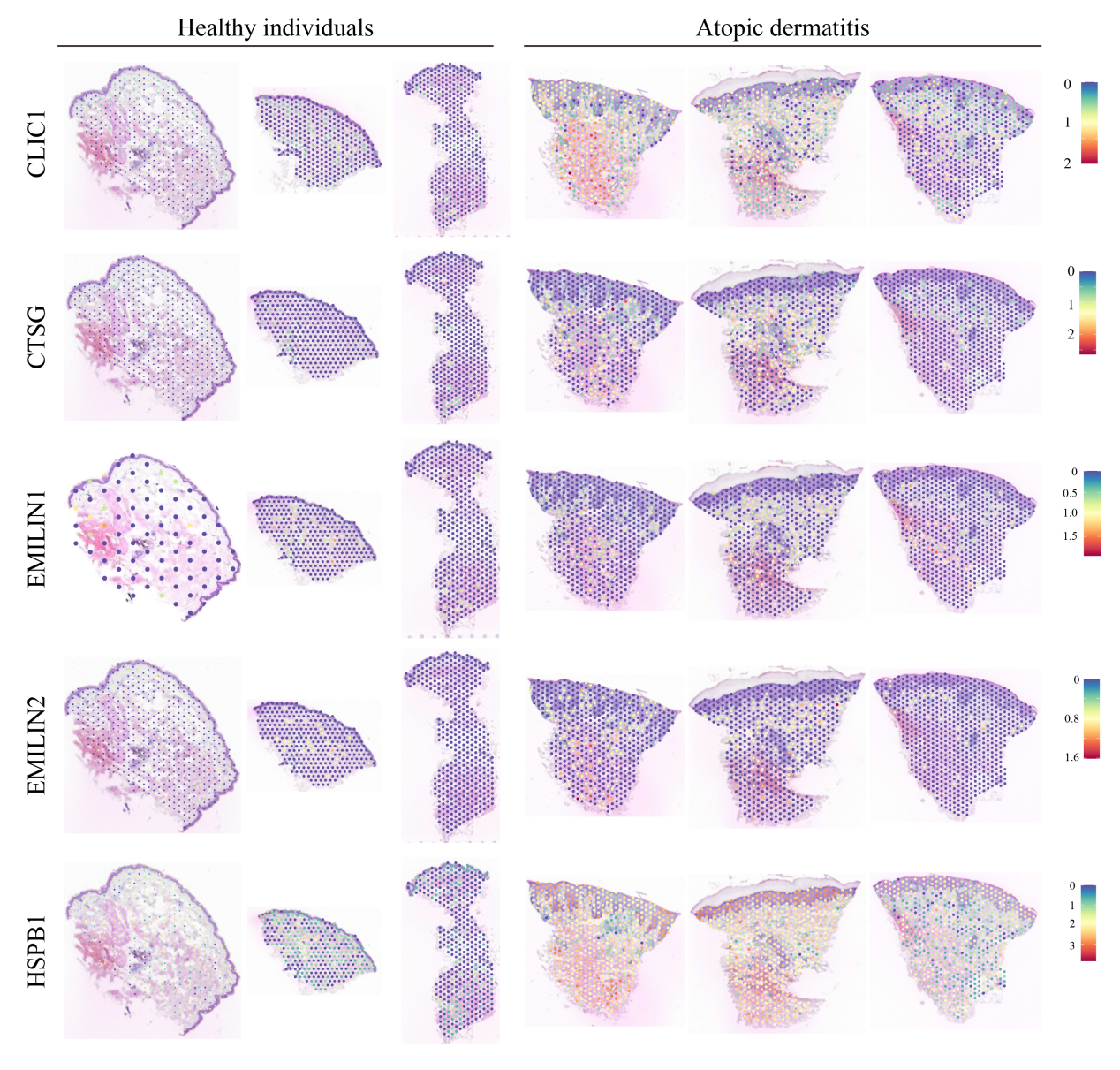


**Fig. S9**. **Platelet aggregation related genes were significantly increased in AD patients**. Representative images of H&E staining and spatial feature plots of platelet aggregation-related genes in skin tissue sections, including *CLIC1*, *CTSG*, *EMILIN1*, *EMILIN2* and *HSPB1.* Spatial transcriptomics data of AD patients were obtained from the GEO database under the accession ID GSE197023 (platform: GPL24676). n = 6 healthy individuals, n = 7 AD patients.


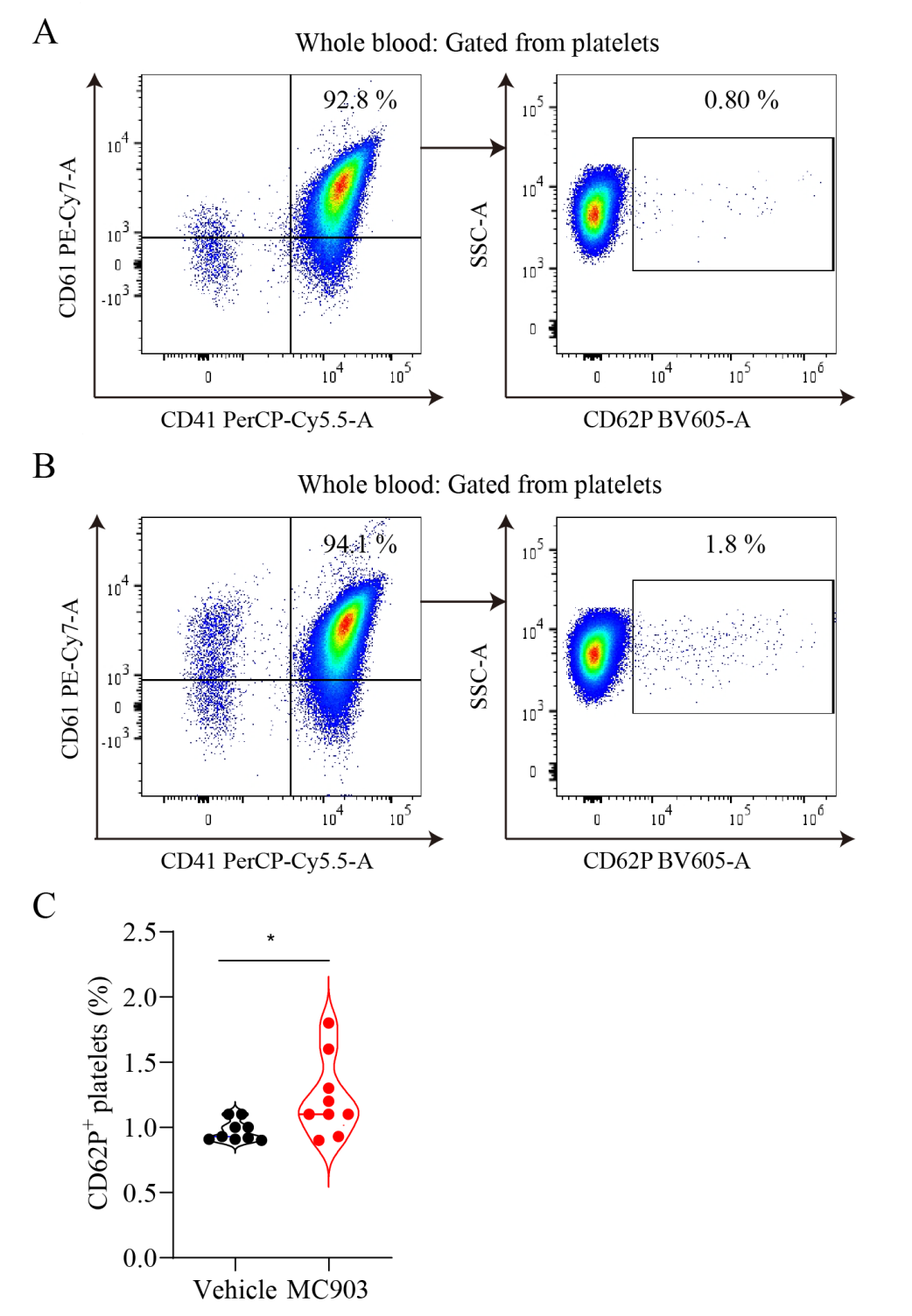


**Fig. S10**. CD62P on platelets is upregulated in MC903-induced AD mice. (A) Representative patterns of expression of CD62P on CD41^+^/CD61^+^ platelets in control mice. (B) Representative expression patterns of CD62P on CD41^+^/CD61^+^ platelets in MC903-induced AD mice. (C) Quantification of CD62P expression on CD41^+^/CD61^+^ platelets in MC903-induced AD mice. n = 9 mice per group. *P* = 0.0285 (two-tailed Mann-Whitney test). **P* < 0.05. Individual data points represent single animals from two independent experiments.


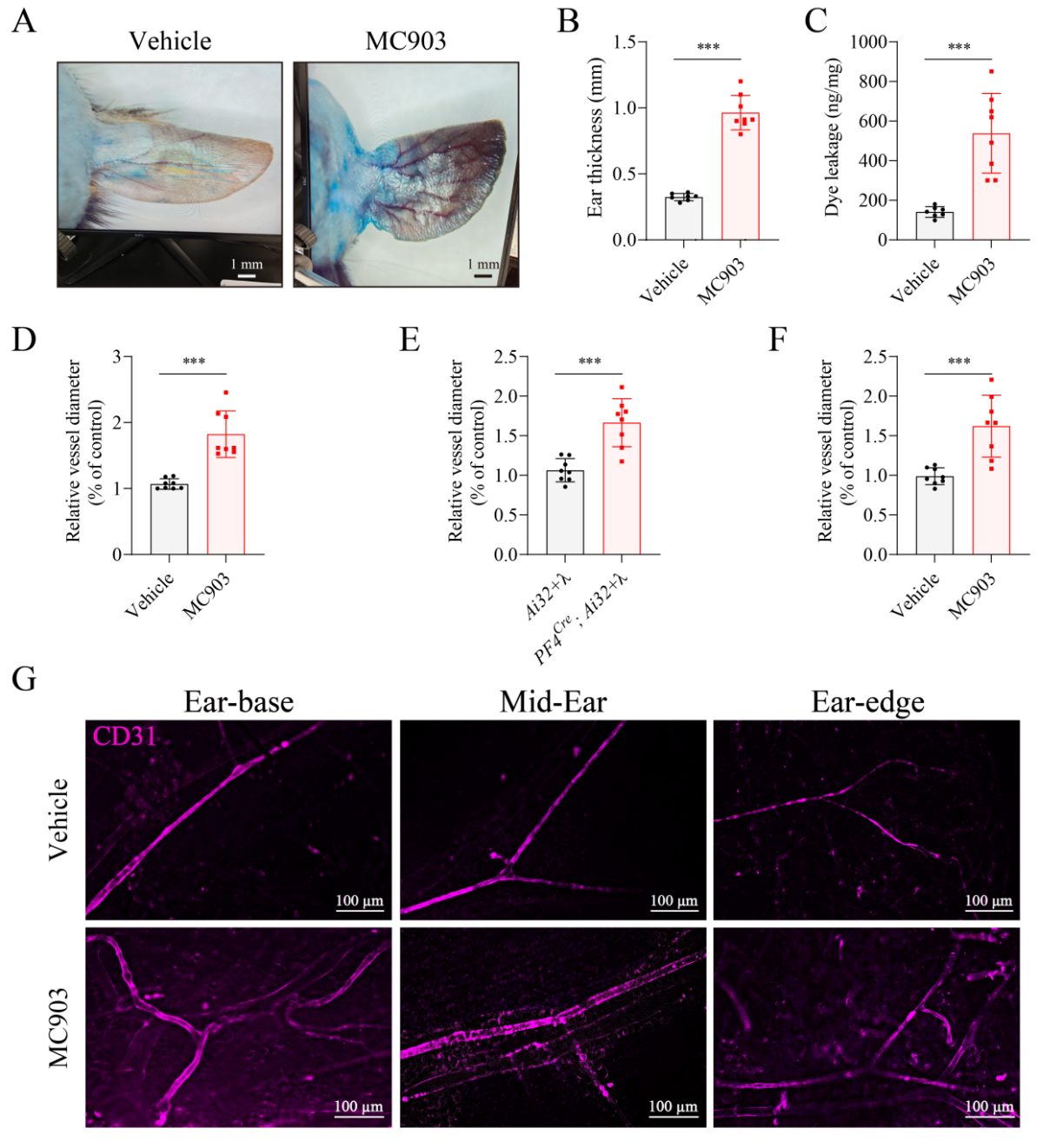


**Fig. S11. Validation of vascular leakage and vasodilation in the ears of MC903-induced AD mice.** (A) Representative photos of Evans blue staining in the ears of vehicle and MC903-treated mice. (B) Quantification of ear thickness in vehicle and MC903-treated mice. n = 7 control mice and n = 8 MC903-induced AD mice. *t_13_* = 12.61, *P* < 0.0001 (two-tailed unpaired Student’s *t* test). (C) Quantification of Evans blue leakage in vehicle and MC903-treated mice. n = 7 control mice and n = 8 MC903-induced AD mice. *t_13_* = 5.154, *P* = 0.0002 (two-tailed unpaired Student’s *t* test). (D-F) Quantification of the vascular diameter at the ear base (D), middle (E) and edge (F) based on CD31 whole mount staining. n = 8 mice per group. For (D), *P* = 0.0002 (two-tailed Mann-Whitney test). For (E), *t_14_* = 5.052, *P* = 0.0002 (two-tailed unpaired Student’s *t* test). For (F), *t_14_* = 4.434, *P* = 0.0006 (two-tailed unpaired Student’s *t* test). (G) Representative images of CD31^+^ vascular in the ear of vehicle and MC903-treated mice. Scale bar, 100 μm. ****P* < 0.001. Individual data points in the quantitative panels represent single animals and are shown as mean ± SD from two independent experiments.

**
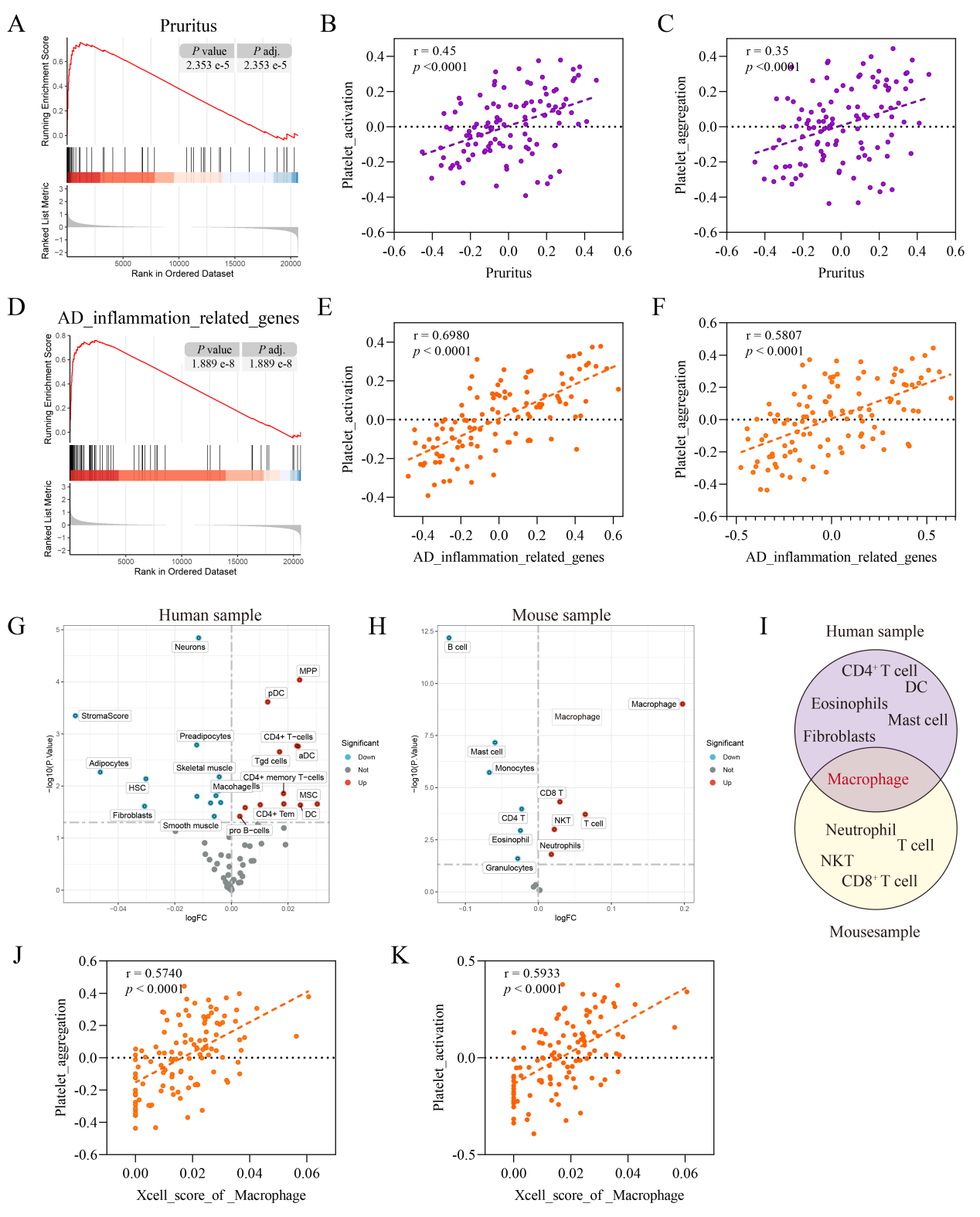
**

**Fig. S12**. **Platelet activation is positively associated with inflammation and itch severity in atopic dermatitis.** (A) GSEA analysis of the pruritus pathways in AD patients. *P* < 0.0001. (B) Correlation analysis of platelet activation and pruritus in AD patients. r = 0.4500, *P* < 0.0001 (two-tailed correlation test). (C) Correlation analysis of platelet aggregation and pruritus in AD patients. r = 0.3500, *P* < 0.0001 (two-tailed correlation test). (D) GSEA analysis of the inflammation-related pathways in AD patients. *P* < 0.0001. (E) Correlation analysis of platelet activation and inflammation-related pathways in AD patients. r = 0.6980, *P* < 0.0001 (two-tailed correlation test). (F) Correlation analysis of platelet aggregation and inflammation-related pathways in AD patients. r = 0.5807, *P* < 0.0001 (two-tailed correlation test). (G-H) Volcano plots showed highly infiltrated immune cells in AD patients (G) and MC903-induced AD mouse model (H). Differentially infiltrated immune cells are defined as adj. *P* < 0.05 and |log_2_FC| > 0. (I) Venn diagram of the highly infiltrated immune cells between AD patients and MC903-induced AD mouse model. (J) Correlation analysis of platelet activation and macrophage infiltration in AD patients. r = 0.5740, *P* < 0.0001 (two-tailed correlation test). (K) Correlation analysis of platelet aggregation and macrophage infiltration in AD patients. r = 0.5933, *P* < 0.0001 (two-tailed correlation test). The RNA-seq data of AD patients were obtained from the GEO database under the accession ID GSE99802 (platform: GPL570). n = 53 healthy individuals, n = 59 AD patients. RNA-seq data of AD mouse model was retrieved from GEO (ID: GSE246569, platform: GPL24247). n = 6 control littermates, n = 8 MC903-induced AD mouse model.


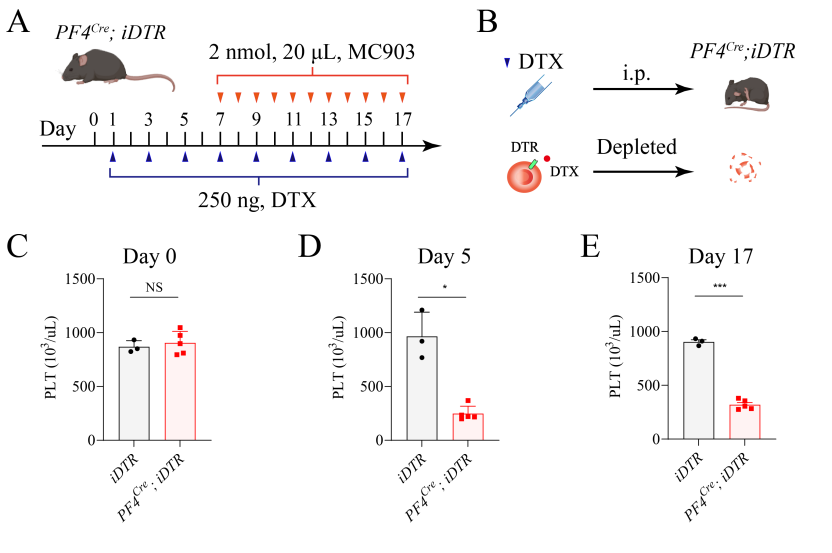


**Fig. S13**. **Selective platelet depletion in *PF4^Cre^; iDTR* mice following DTX administration.** (A) Schematic of pharmacological depletion of platelets using DTX following MC903-induced AD mouse modeling. (B) The mechanism of the pharmacological depletion of platelets through DTX administration. (C-E) Changes in platelet count in whole blood after DTX administration on day 0 (C), day 5 (D) and day 17 (E). For (D), *P* = 0.0357 (two-tailed Mann-Whitney test). For (E), *t_6_* = 19.20, *P* < 0.0001 (two-tailed unpaired Student’s *t* test). n = 3 *iDTR* mice, n = 4 *PF4^Cre^; iDTR* mice. **P* < 0.05, ***P* < 0.01, NS, not significant. Individual data points in the quantitative panels represent single animals and are shown as mean ± SD from one representative of two independent experiments with consistent results.


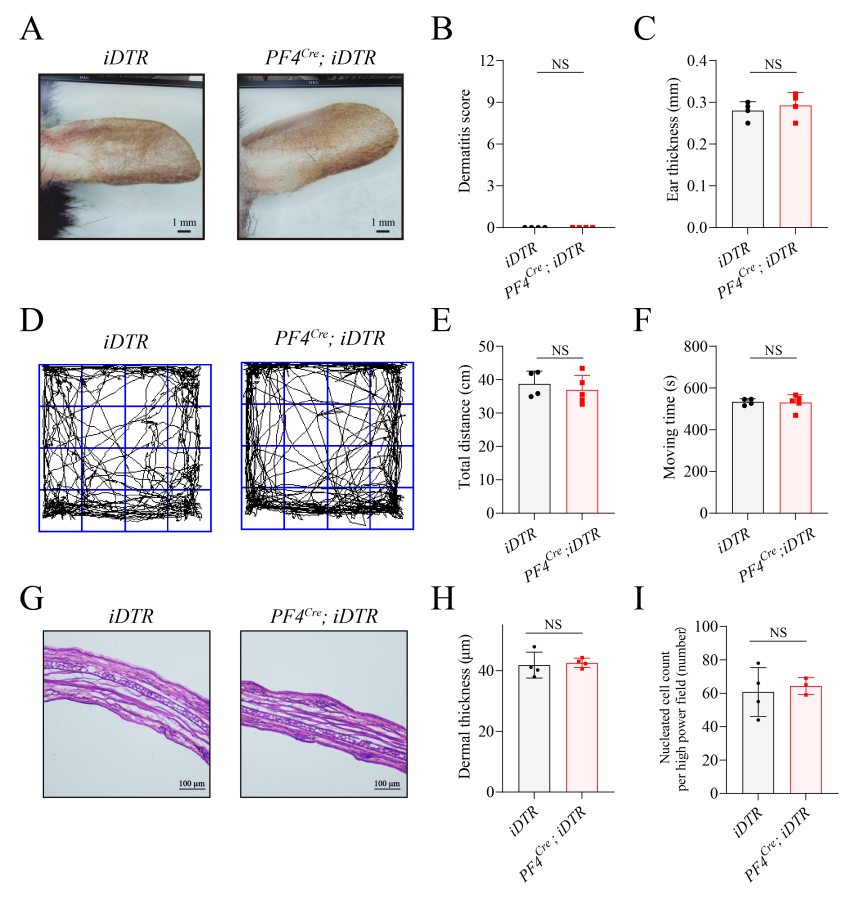


**Fig. S14.** **Depletion of platelets does not alter baseline skin morphology or spontaneous behavior.** (A) Representative clinical images of ears from DTX-treated *iDTR* control littermates and *PF4^Cre^; iDTR* mice under basal conditions. Scale bar, 1 mm. (B-C) Quantification of dermatitis scores (B) and ear thickness (C). n = 4 mice per group (two-tailed unpaired Student’s *t* test). (D) Representative open-field test results from DTX-treated *iDTR* control littermates and *PF4^Cre^; iDTR* mice under basal conditions. (E-F) Quantification of total distance (E) and moving time (F). n = 5 mice per group (two-tailed unpaired Student’s *t* test). (G) Representative H&E-stained skin sections from DTX-treated *iDTR* control littermates and *PF4^Cre^; iDTR* mice under basal conditions. n = 3-5 sections from 3 mice. (H-I) Quantification of epidermal thickness (H) and dermal immune cell infiltration (I). n = 4 mice per group (two-tailed unpaired Student’s *t* test). NS, not significant. Individual data points in the quantitative panels represent single animals and are shown as mean ± SD from one representative of two independent experiments with consistent results.


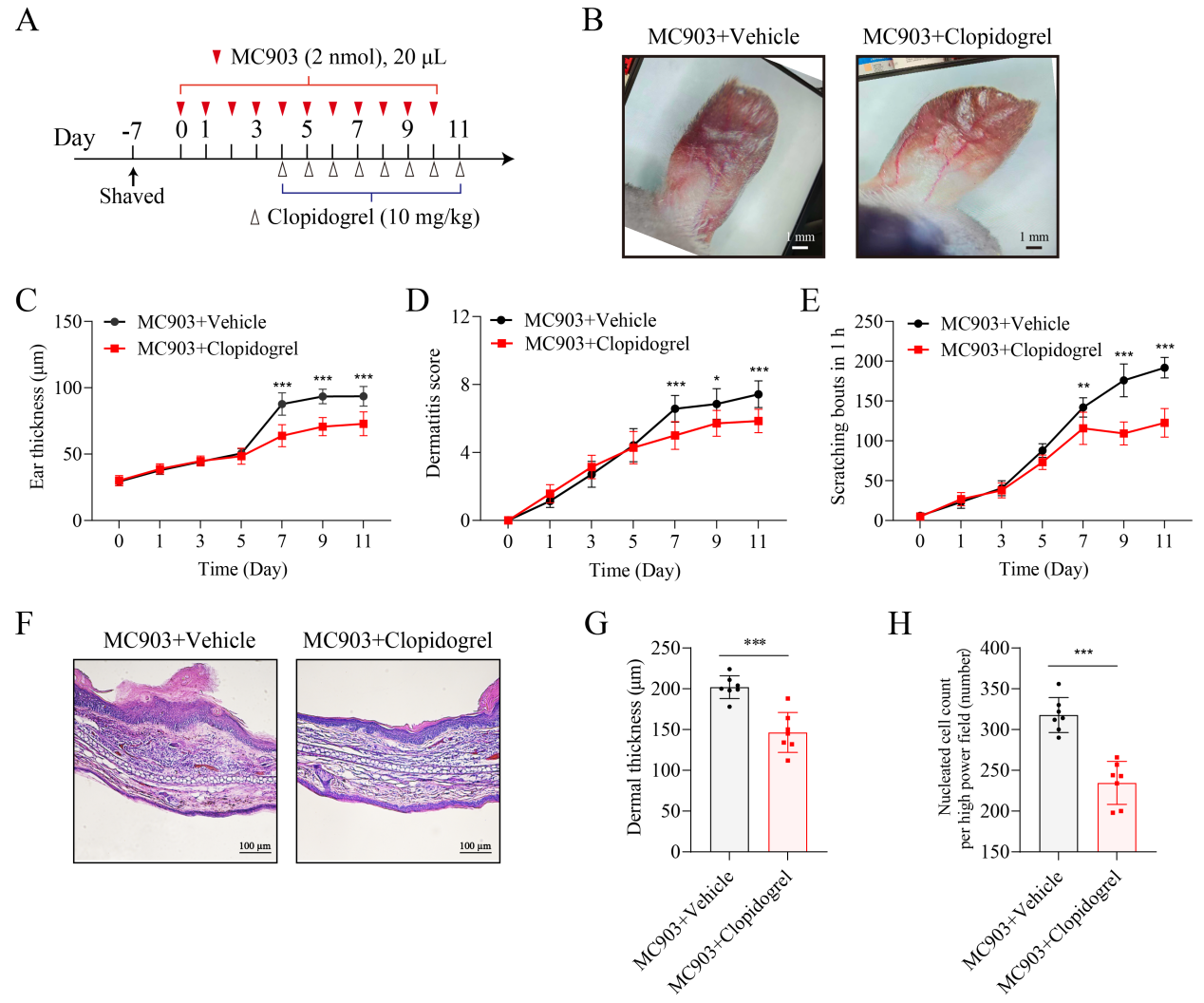


**Fig. S15. Clopidogrel suppressed MC903 induced cutaneous inflammation and pruritus.** (A) Schematic of the clopidogrel treatment for MC903 induced AD mouse model. (B) Representative images of ears. Scale bar, 1 mm. (C-D) Ear thickness (C) and inflammation score (D) measured at the indicated time points. n = 7 mice per group. For (C), *F_6,84_* = 15.26, *P* < 0.0001. day 9: *P* < 0.0001, day 11: *P* < 0.0001. For (D), *F_6,84_* = 5.537, *P* < 0.0001. day 7: *P* = 0.0006, day 9: *P* = 0.0244; day 11: *P* = 0.0006 (two-way ANOVA followed by Šídák’s multiple comparisons test). (E) Quantification of spontaneous scratching bouts. n = 6 mice per group. *F_6,84_* = 21.52, *P* < 0.0001. day 7: *P* = 0.0011, day 9: *P* < 0.0001, day 11: *P* < 0.0001 (two-way ANOVA followed by Šídák’s multiple comparisons test). (F) Representative H&E histopathology images of ears. n = 3-5 sections from 3 mice. Scale bar, 100 μm. (G-H) Quantification of dermal thickness (G) and dermal immune cell infiltration (H). n = 7 mice per group. For (G), *t_12_* = 5.213, *P* = 0.0002. For (H), *t_12_* = 6.465, *P* < 0.0001 (two-tailed unpaired Student’s *t* test). **P* < 0.05, ***P* < 0.01, ****P* < 0.001. Individual data points in the quantitative panels represent single animals and are shown as mean ± SD from two independent experiments.


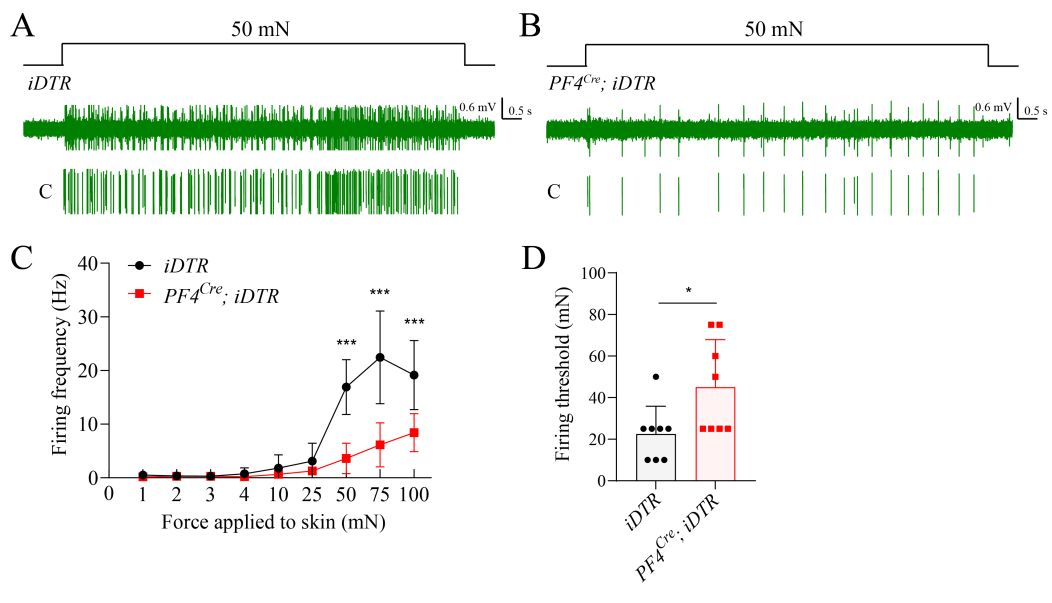


**Fig. S16**. **Platelet-depleted mice exhibited a diminished response to mechanical stimuli.** (A-B) Representative traces of mechanically evoked C fiber firings in response to 50 mN force recorded *ex vivo* from control littermates (A) and platelet-depleted *PF4^Cre^; iDTR* mice (B). (C-D) Summary data showing mechanically evoked C fiber firing frequency (C) and mechanical firing threshold (D). n = 8 units from 4 *iDTR* mice, n = 7 units from 4 *PF4^Cre^; iDTR* mice. For (C), *F_8,117_* = 13.91, *P* < 0.0001. 50 mN: *P* < 0.0001, 75 mN: *P* < 0.0001, 100 mN: *P* < 0.0001 (two-way ANOVA followed by Šídák’s multiple comparisons test). For (D), *P* = 0.0305 (two-tailed Mann-Whitney test). **P* < 0.05, ****P* < 0.001. Individual data points in (C-D) represent single units, and bars show mean ± SD from two independent experiments.


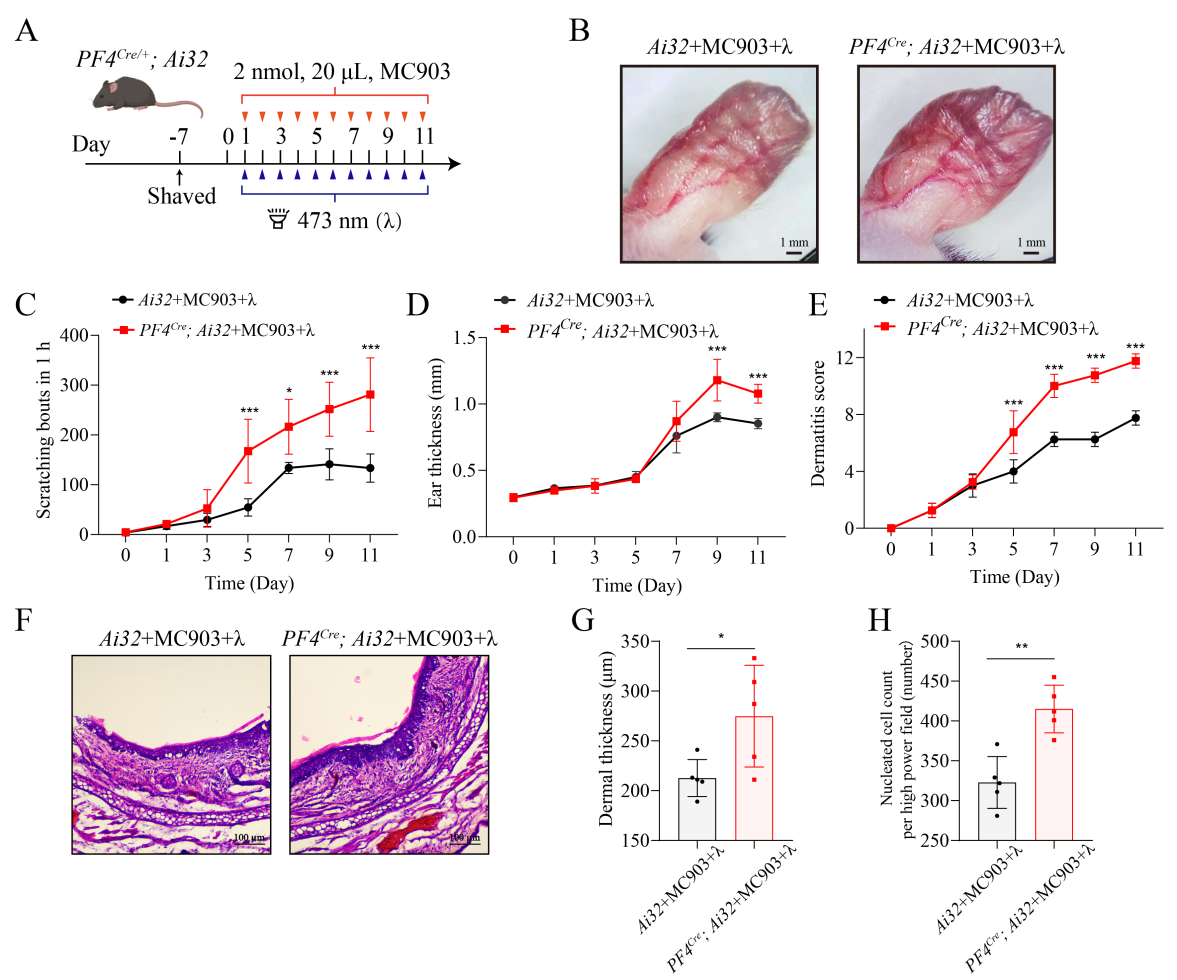


**Fig. S17**. **Optogenetic activation of platelets exacerbated cutaneous inflammation and pruritus in AD model.** (A) Schematic of photostimulation (+λ) of platelets in *Ai32* and *PF4^Cre^; Ai32* mice following MC903-induced AD mouse modeling. (B) Representative images of ears. Scale bar, 1 mm. (C) Quantification of spontaneous scratching bouts. n = 4 mice per group. *F_8,42_* = 5.046, *P* = 0.0006. day 3: *P* = 0.0007, day 5: *P* = 0.0212, day 7: *P* = 0.0009, day 9: *P* < 0.0001, day 11: *P* = 0.0467 (two-way ANOVA followed by Šídák’s multiple comparisons test). (D-E) Ear thickness (D) and inflammation score (E) measured at the indicated time points. n = 4 mice per group. For (D), *F_6,42_* = 5.607, *P* = 0.0002. day 9: *P* < 0.0001, day 11: *P* = 0.0008. For (E), *F_6,42_* = 18.45, *P* < 0.0001. day 5: *P* < 0.0001, day 7: *P* < 0.0001, day 9: *P* < 0.0001, day 11: *P* < 0.0001 (two-way ANOVA followed by Šídák’s multiple comparisons test). (F) Representative H&E histopathology images of ears. n = 3-5 sections from 3 mice. Scale bar, 100 μm. (G-H) Quantification of dermal thickness (G) and dermal immune cell infiltration (H). n = 5 mice per group. For (G), *t_8_* = 2.559, *P* = 0.0337. For (H), *t_8_* = 4.660, *P* = 0.0016 (two-tailed unpaired Student’s *t* test). **P* < 0.05, ***P* < 0.01, ****P* < 0.001. Individual data points in the quantitative panels represent single animals and are shown as mean ± SD from one representative of two independent experiments with consistent results.


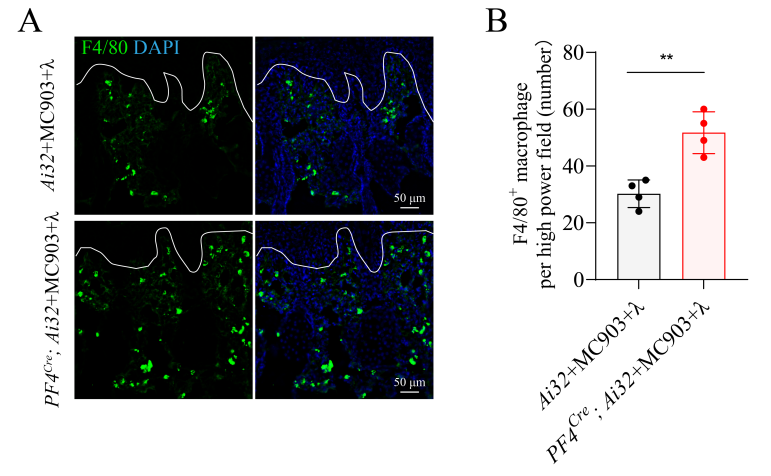


**Fig. S18**. **Optogenetic platelet activation is associated with enhanced macrophage infiltration in AD.** (A) Representative images of F4/80^+^ macrophages in the skin of the photostimulated *Ai32* and *PF4^Cre^; Ai32* mice following MC903 treatment, which were quantified in (B). Scale bar, 50 μm. n = 4 mice per group. For (B), *t_6_* = 4.874, *P* = 0.0028 (two-tailed unpaired Student’s *t* test). ***P* < 0.01. Individual data points in the quantitative panels represent single animals and are shown as mean ± SD from one representative of two independent experiments with consistent results.


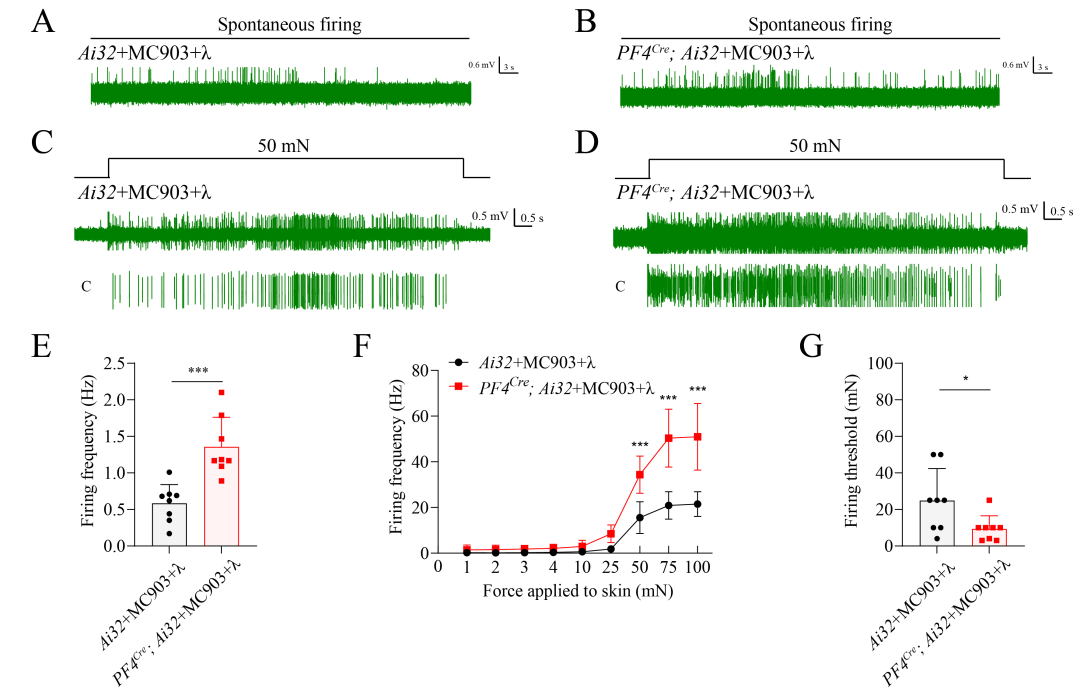


**Fig. S19**. **Optogenetic activation of platelets markedly increased C-fiber excitability in AD.** (A-B) Representative traces of spontaneous C fiber firings recorded *ex vivo* from *Ai32* control (A) and *PF4^Cre^; Ai32* (B) mice following MC903-induced AD mouse modeling. (C-D) Representative traces of mechanically evoked C fiber firings in response to 50 mN force. (E-G) Summary data showing spontaneous C fiber firing frequency (E), mechanically evoked C fiber firing frequency (F), and mechanical firing threshold (G). n = 6-8 units from 4 mice. For (E), *P* = 0.0003 (two-tailed Mann-Whitney test). For (F), *F_8,90_* = 13.55, *P* < 0.0001. 50 mN: *P* < 0.0001, 75 mN: *P* < 0.0001, 100 mN: *P* < 0.0001 (two-way ANOVA followed by Šídák’s multiple comparisons test). For (G), *P* = 0.0435 (two-tailed Mann-Whitney test). **P* < 0.05, ****P* < 0.001. Individual data points in (E-G) represent single units, and bars show mean ± SD from two independent experiments.


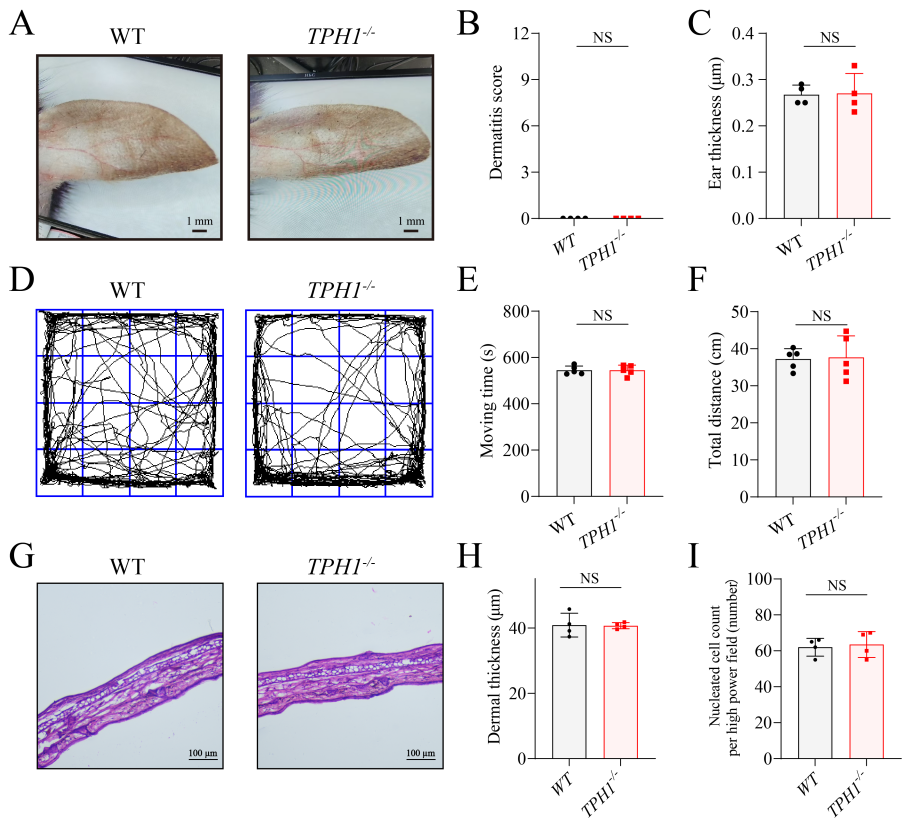


**Fig. S20.** **Depletion of platelet-derived serotonin does not alter baseline skin morphology or spontaneous behavior.** (A) Representative clinical images of ears from WT and *TPH1^-/-^* mice under basal conditions. Scale bar, 1 mm. (B-C) Quantification of dermatitis scores (B) and ear thickness (C). n = 4 mice per group (two-tailed unpaired Student’s *t* test). (D) Representative open-field test results from WT and *TPH1^-/-^* mice under basal conditions. (E-F) Quantification of total distance (E) and moving time (F). n = 5 mice per group (two-tailed unpaired Student’s *t* test). (G) Representative H&E-stained skin sections from WT and *TPH1^-/-^* mice under basal conditions. n = 3-5 sections from 3 mice. (H-I) Quantification of epidermal thickness (H) and dermal immune cell infiltration (I). n = 4 mice per group (two-tailed unpaired Student’s *t* test). NS, not significant. Individual data points in the quantitative panels represent single animals and are shown as mean ± SD from one representative of two independent experiments with consistent results.


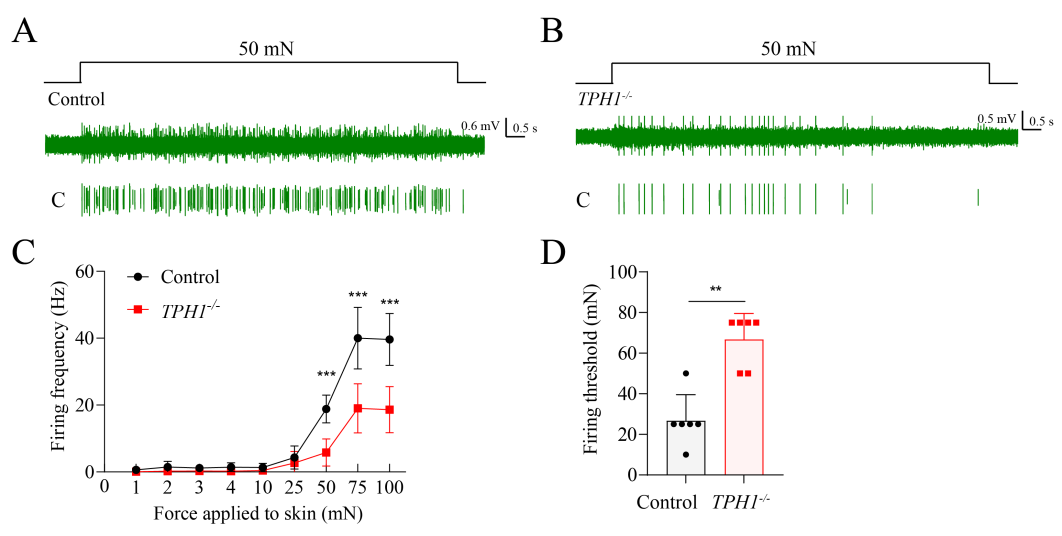


**Fig. S21**. **Depletion of platelet-derived serotonin reduced the mechanically evoked C fiber firings to mechanical stimuli.** (A-B) Representative traces of mechanically evoked C fiber firings in response to 50 mN force recorded *ex vivo* from the control littermates (A) and *TPH1^-/-^* mice (B). (C-D) Summary data showing mechanically evoked C fiber firing frequency (C) and mechanical firing threshold (D). n = 6-7 units from 4 mice. For (C), *F_8,99_* = 15.08, *P* < 0.0001. 50 mN: *P* < 0.0001, 75 mN: *P* < 0.0001, 100 mN: *P* < 0.0001 (two-way ANOVA followed by Šídák’s multiple comparisons test). For (D), *P* = 0.0065 (two-tailed Mann-Whitney test). ***P* < 0.01, ****P* < 0.001. Individual data points in (C-D) represent single units, and bars show mean ± SD from two independent experiments.


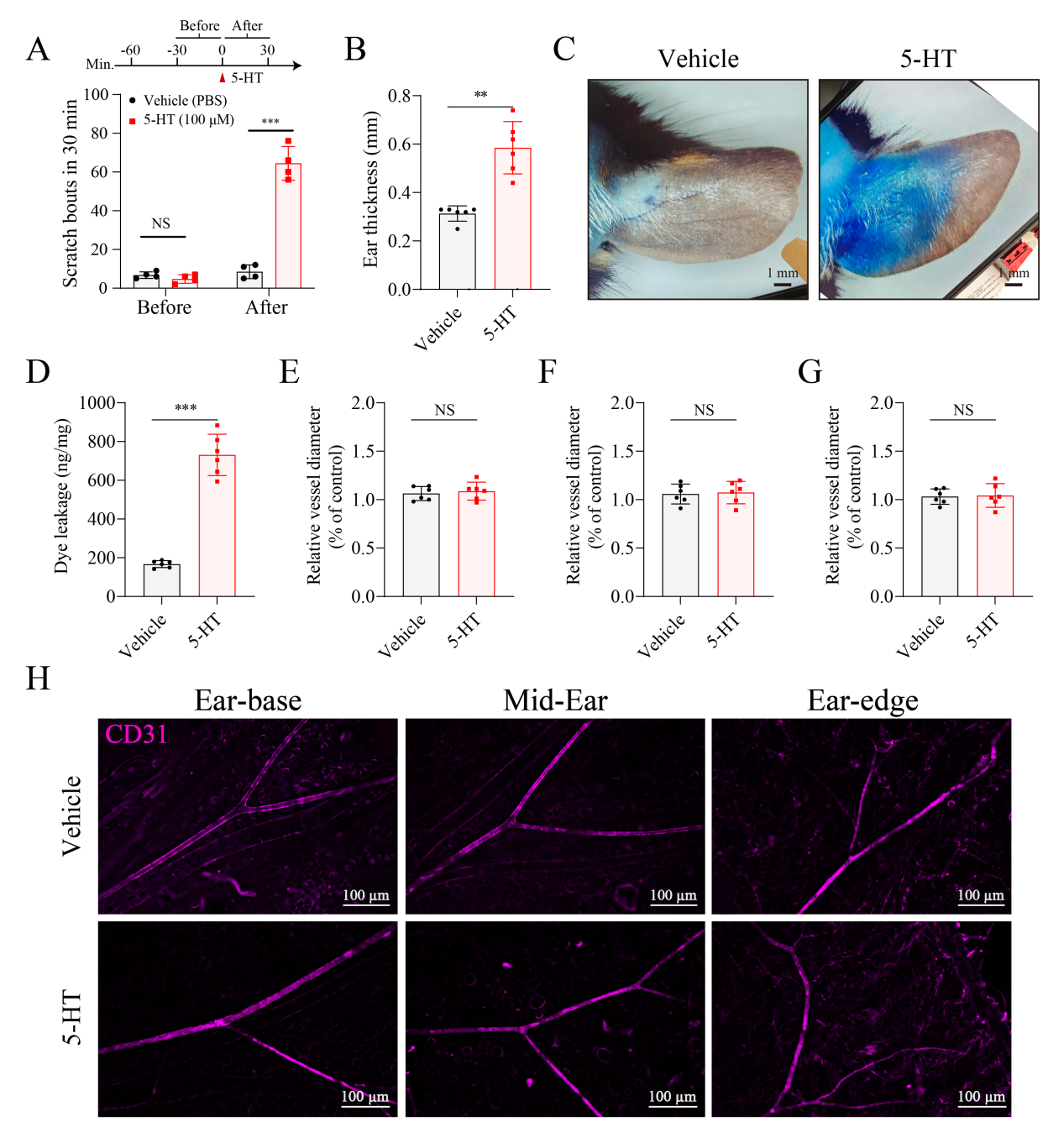


**Fig. S22. Validation of vascular leakage and vasodilation in the ears of the acute 5-HT injection.** (A) Quantification of spontaneous scratching bouts following the 5-HT (100 μM, 20 μL) or vehicle (PBS, 20 μL) treatment. n = 4 mice per group. *t_6_* = 11.94, *P* < 0.0001 (two-tailed unpaired Student’s *t* test). (B) Quantification of ear thickness. n = 6 mice per group. *P* = 0.0022 (two-tailed Mann-Whitney test). (C) Representative photos of Evans blue staining in the ears. (D) Quantification of Evans blue leakage in vehicle and 5-HT treated mice. n = 6 mice per group. *t_10_* = 12.79, *P* < 0.0001 (two-tailed unpaired Student’s *t* test). (E-G) Quantification of the vascular diameter at the ear base (E), middle (F) and edge (G) based on CD31 whole mount staining. n = 6 mice per group. (H) Representative images of CD31^+^ vascular in the ear of vehicle and 5-HT treated mice. Scale bar, 100 μm. ***P* < 0.01, ****P* < 0.001. Individual data points in the quantitative panels represent single animals and are shown as mean ± SD from two independent experiments.

**
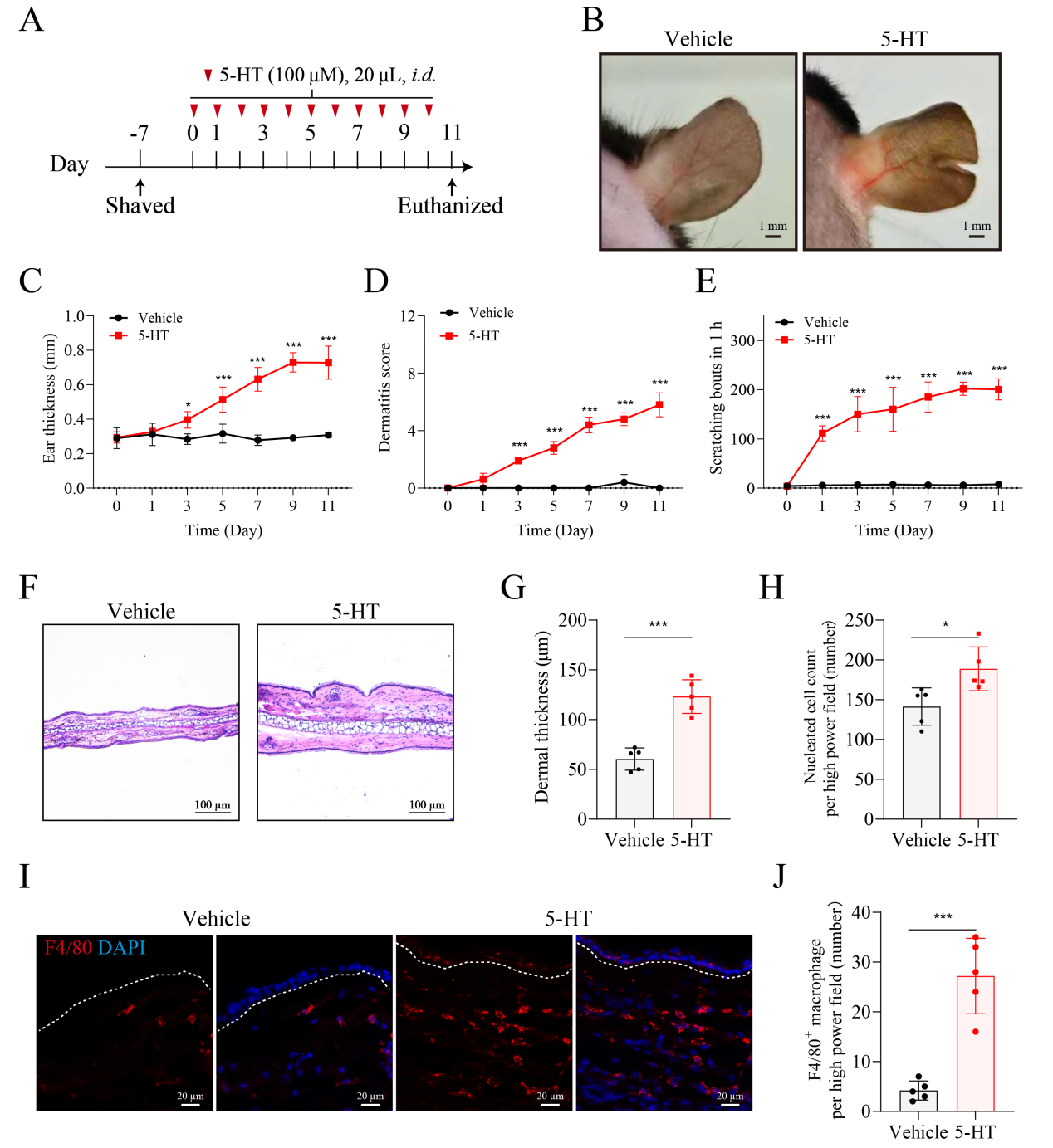
**

**Fig. S23.** **5-HT induced pruritus and skin inflammation.** (A) Schematic illustration of 5-HT treatment. (B) Representative images of ears from vehicle- or 5-HT-treated mice. Scale bar, 1 mm. (C-D) Ear thickness (C) and inflammation score (D) measured at the indicated time points. n = 5 mice per group. For (C), *F_6, 56_* = 30.61, *P* < 0.0001. day 3: *P* = 0.0105, day 5: *P* < 0.0001, day 7: *P* < 0.0001, day 9: *P* < 0.0001, day 11: *P* < 0.0001. For (D), *F_6, 56_* = 83.49, *P* < 0.0001. day 3: *P* < 0.0001, day 5: *P* < 0.0001, day 7: *P* < 0.0001, day 9: *P* < 0.0001, day 11: *P* < 0.0001 (two-way ANOVA followed by Šídák’s multiple comparisons test). (E) Quantification of spontaneous scratching bouts. n = 6 mice per group. *F_6, 56_* = 32.05, *P* < 0.0001. day 1: *P* < 0.0001, day 3: *P* < 0.0001, day 5: *P* < 0.0001, day 7: *P* < 0.0001, day 9: *P* < 0.0001, day 11: *P* < 0.0001 (two-way ANOVA followed by Šídák’s multiple comparisons test). (F) Representative H&E histopathology images of ear sections. n = 3-5 sections from 3 mice. Scale bar, 100 μm. (G-H) Quantification of dermal thickness (G) and dermal immune cell infiltration (H). n = 6 mice per group. For (G), *t_8_* = 6.927, *P* = 0.0001. For (H), *t_8_* = 2.935, *P* = 0.0189 (two-tailed unpaired Student’s *t* test). (I-J) Representative images of F4/80^+^ macrophages in the skin of vehicle and 5-HT-treaated mice, which were quantified in (J). Scale bar, 20 μm. n = 5 mice per group. For (J), *t_8_* = 6.563, *P* = 0.0002 (two-tailed unpaired Student’s *t* test). **P* < 0.05, ***P* < 0.01, ****P* < 0.001. Individual data points in the quantitative panels represent single animals and are shown as mean ± SD from two independent experiments with consistent results.


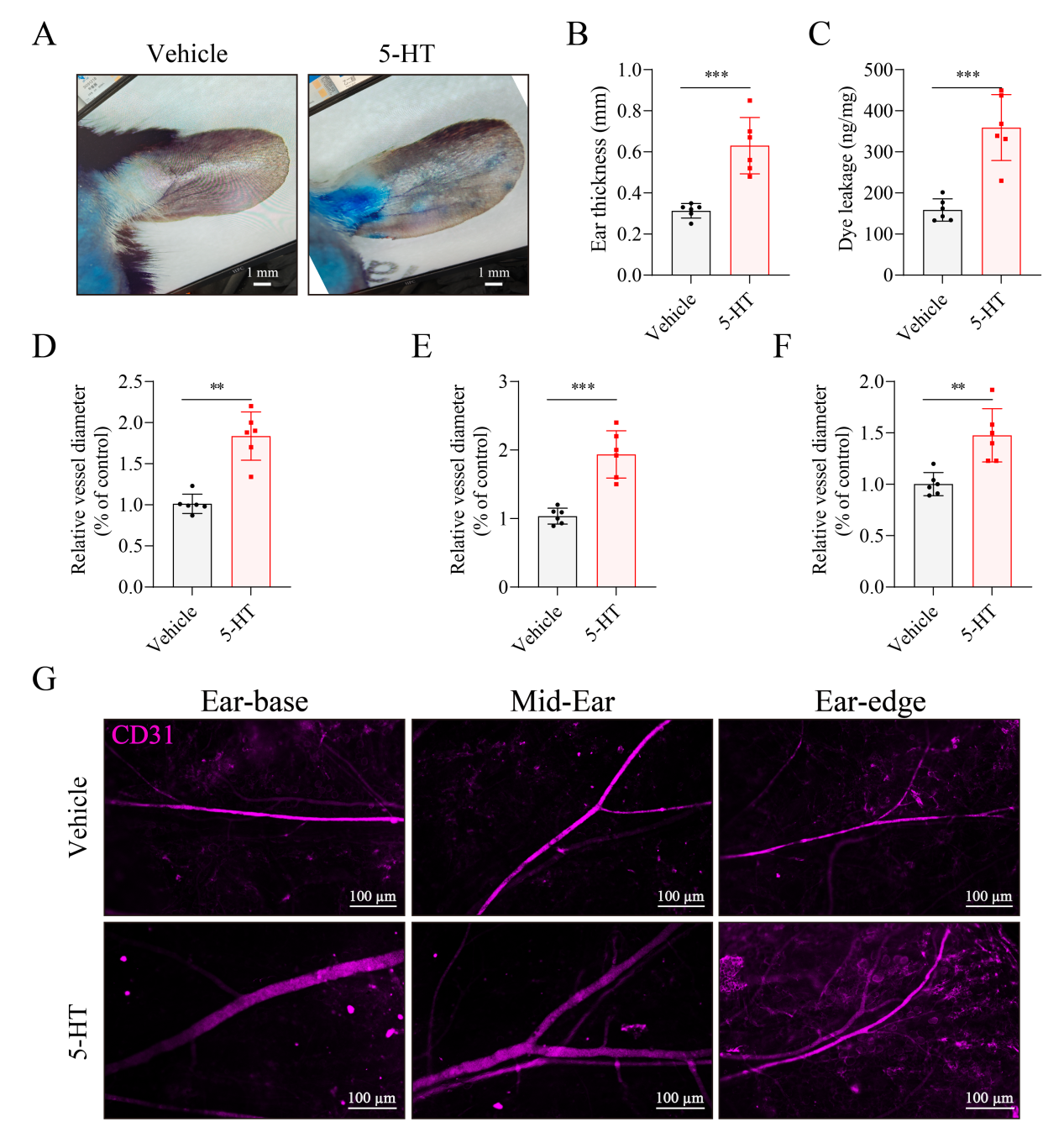


**Fig. S24. Validation of vascular leakage and vasodilation in the ears of the chronic 5-HT injection.** (A) Representative photos of Evans blue staining in the ears. (B) Quantification of ear thickness. n = 6 mice per group. *t_10_* = 5.470, *P* = 0.0003 (two-tailed unpaired Student’s *t* test). (C) Quantification of Evans blue leakage in vehicle and 5-HT treated mice. n = 6 mice per group. *t_10_* = 5.805, *P* = 0.0002 (two-tailed unpaired Student’s *t* test). (D-F) Quantification of the vascular diameter at the ear base (D), middle (E) and edge (F) based on CD31 whole mount staining. n = 6 mice per group. For (D), *P* = 0.0022 (two-tailed Mann-Whitney test). For (E), *t_10_* = 6.079, *P* = 0.0001 (two-tailed unpaired Student’s *t* test). For (F), *P* = 0.0022 (two-tailed Mann-Whitney test). (G) Representative images of CD31^+^ vascular in the ears of vehicle and 5-HT treated mice. Scale bar, 100 μm. ***P* < 0.01, ****P* < 0.001. Individual data points in the quantitative panels represent single animals and are shown as mean ± SD from two independent experiments.


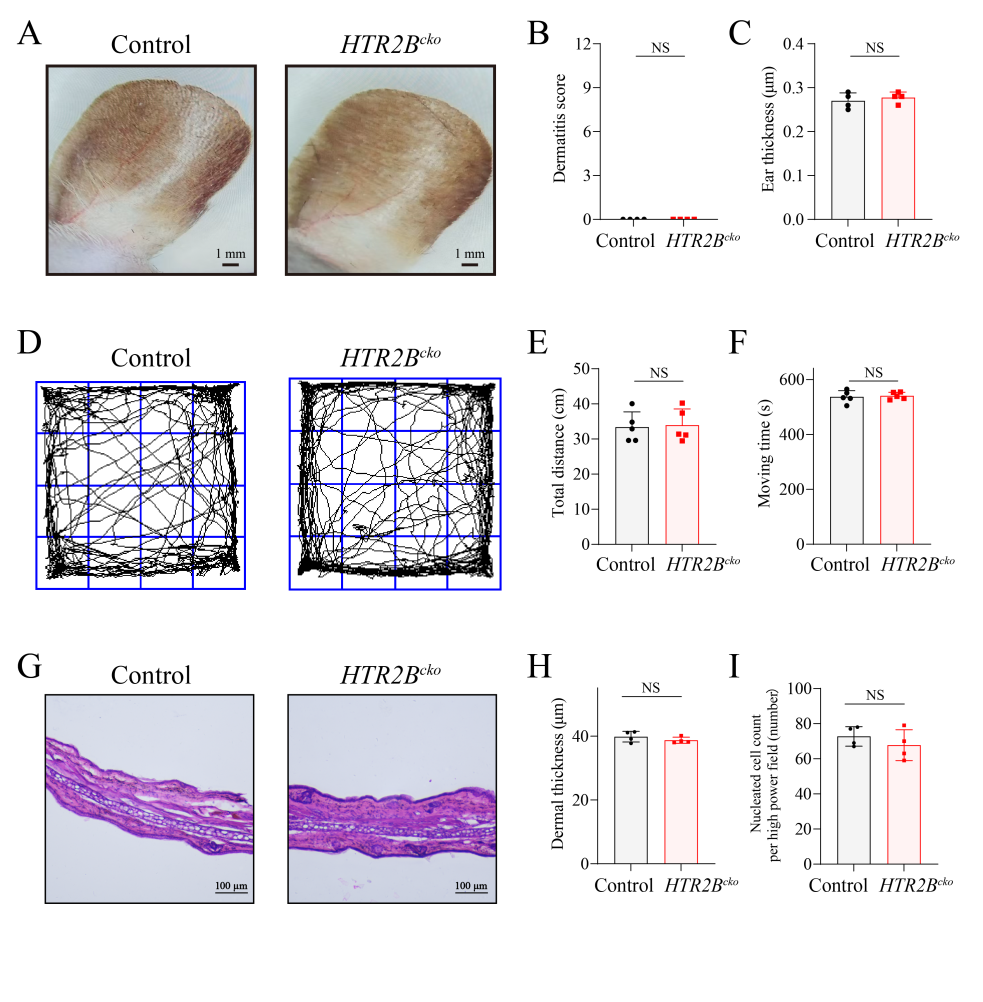


**Fig. S25.** **TRPV1-neuron-specific deletion of HTR2B does not alter baseline skin morphology or behavior.** (A) Representative clinical images of ears from control littermates and *HTR2B^cko^* mice under basal conditions. Scale bar, 1 mm. (B-C) Quantification of dermatitis scores (B) and ear thickness (C). n = 4 mice per group (two-tailed unpaired Student’s *t* test). (D) Representative open-field test results from control littermates and *HTR2B^cko^* mice under basal conditions. (E-F) Quantification of total distance (E) and moving time (F). n = 5 mice per group (two-tailed unpaired Student’s *t* test). (G) Representative H&E-stained skin sections from control littermates and *HTR2B^cko^* mice under basal conditions. n = 3-5 sections from 3 mice. (H-I) Quantification of epidermal thickness (H) and dermal immune cell infiltration (I). n = 4 mice per group (two-tailed unpaired Student’s *t* test). NS, not significant. Individual data points in the quantitative panels represent single animals and are shown as mean ± SD from one representative of two independent experiments with consistent results.

**
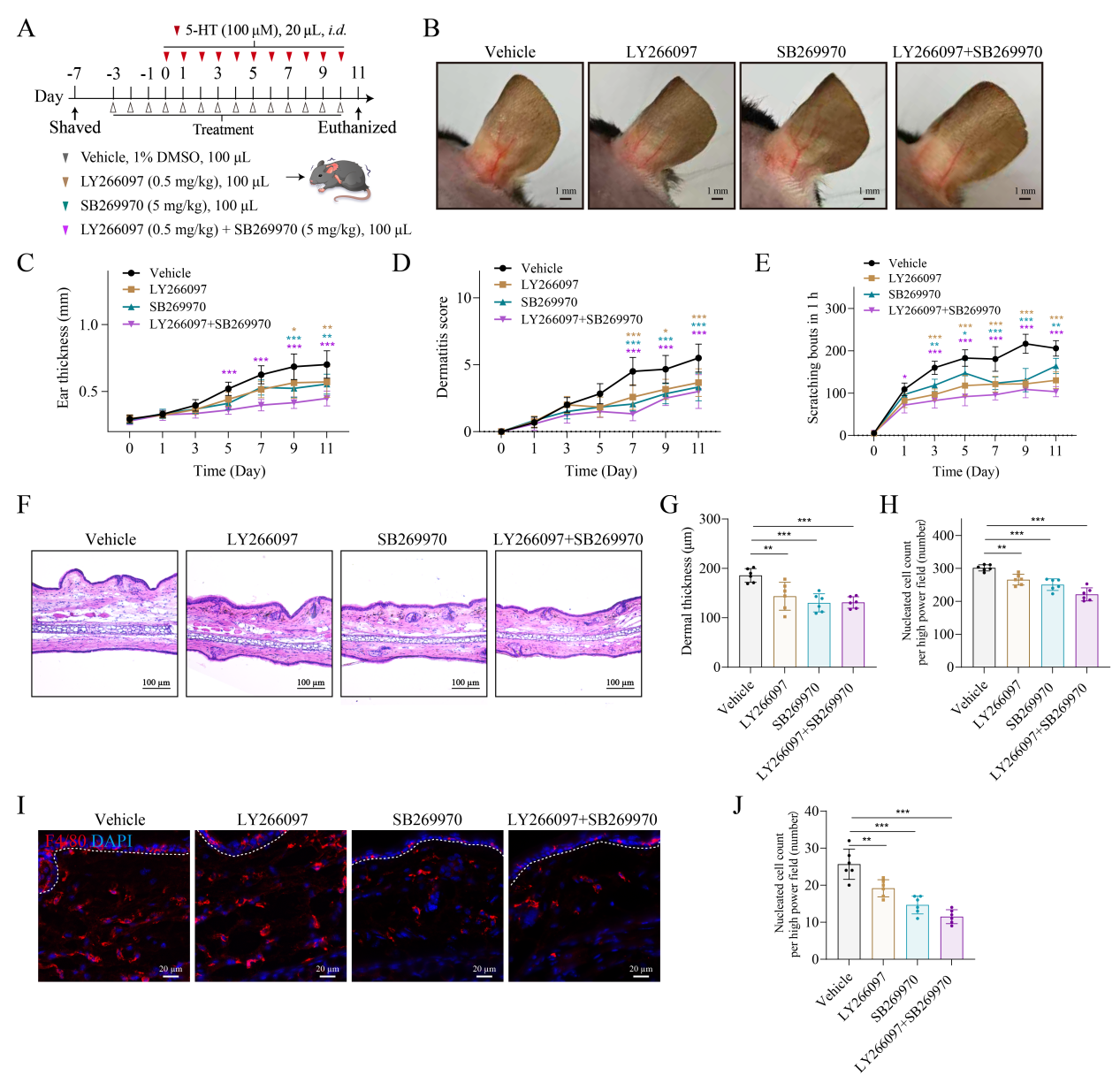
**

**Fig. S26.** **HTR antagonist treatment attenuates pruritus and skin inflammation induced by intradermal 5-HT injection.** (A) Schematic illustration of HTR2B inhibitor (LY266097) and/or HTR7 inhibitor (SB269970) applications in 5-HT-treated mice. (B) Representative images of ears from 5-HT-treated mice after 11 days of HTR2B inhibitor and/or HTR7 inhibitor applications. Scale bar, 1 mm. (C-D) Ear thickness (C) and inflammation score (D) measured at the indicated time points. n = 6 mice per group. For (C), *F_18,140_* = 3.994, *P* < 0.0001. Vehicle *vs*. LY266097: day 9: *P* = 0.0204, day 11: *P* = 0.0070. Vehicle *vs*. SB269970: day 9: *P* = 0.0002, day 11: *P* = 0.0010. Vehicle *vs*. LY266097+SB269970: day 5: *P* = 0.0002, day 7: *P* < 0.0001, day 9: *P* < 0.0001, day 11: *P* < 0.0001. For (D), *F_18,140_* = 3.761, *P* < 0.0001. Vehicle *vs*. LY266097: day 7: *P* = 0.0002, day 9: *P* = 0.0124, day 11: *P* = 0.0005. Vehicle *vs*. SB269970: day 7: *P* < 0.0001, day 9: *P* = 0.0005, day 11: *P* < 0.0001. Vehicle *vs*. LY266097+SB269970: day 7: *P* < 0.0001, day 9: *P* < 0.0001, day 11: *P* < 0.0001 (two-way ANOVA followed by Šídák’s multiple comparisons test). (E) Quantification of spontaneous scratching bouts. n = 6 mice per group. *F_18,140_* = 6.449, *P* < 0.0001. Vehicle *vs*. LY266097: day 3: *P* < 0.0001, day 5: *P* < 0.0001, day 7: *P* < 0.0001, day 9: *P* < 0.0001, day 11: *P* < 0.0001. Vehicle *vs*. SB269970: day 3: *P* = 0.0036, day 5: *P* = 0.0267, day 7: *P* < 0.0001, day 9: *P* < 0.0001, day 11: *P* = 0.0034. Vehicle *vs*. LY266097+SB269970: day 1: *P* = 0.0152, day 3: *P* < 0.0001, day 5: *P* < 0.0001, day 7: *P* < 0.0001, day 9: *P* < 0.0001, day 11: *P* < 0.0001 (two-way ANOVA followed by Šídák’s multiple comparisons test). (F) Representative H&E histopathology images of ear sections. n = 3-5 sections from 3 mice. Scale bar, 100 μm. (G-H) Quantification of dermal thickness (G) and dermal immune cell infiltration (H). n = 6 mice per group. For (G), *F_3,20_* = 11.23, *P* = 0.0002. Vehicle *vs*. LY266097: *P* = 0.0032. Vehicle *vs*. SB269970: *P* = 0.0002. Vehicle *vs*. LY266097+SB269970: *P* = 0.0002. For (H), *F_3,20_* = 25.42, *P* < 0.0001. Vehicle *vs*. LY266097: *P* = 0.0033. Vehicle *vs*. SB269970: *P* < 0.0001. Vehicle *vs*. LY266097+SB269970: *P* < 0.0001 (one-way ANOVA followed by Holm-Šídák’s multiple comparisons test). (I-J) Representative images of F4/80^+^ macrophages in the skin, which were quantified in (J). Scale bar, 20 μm. n = 6 mice per group. For (J), *F_3,20_* = 28.85, *P* < 0.0001. Vehicle *vs*. LY266097: *P* = 0.0020. Vehicle *vs*. SB269970: *P* < 0.0001. Vehicle *vs*. LY266097+SB269970: *P* < 0.0001 (one-way ANOVA followed by Holm-Šídák’s multiple comparisons test). **P* < 0.05, ***P* < 0.01, ****P* < 0.001. Individual data points in the quantitative panels represent single animals and are shown as mean ± SD from two independent experiments.

**Table S1. List of primers used for RT-qPCR.**

| **Target gene** | **Forward primers** | **Reverse primers** |  |
| --- | --- | --- | --- |
| mouse-*GAPDH* | CCTCGTCCCGTAGACAAAATG | TGAGGTCAATGAAGGGGTCGT | |
| mouse-*5Htr1a* | GGCTCATTGGCTTTCTCATCTC | TGGTGTACCCGTGGTCCTTG | |
| mouse-*5Htr1b* | TTCTATTTACCCACCCTGCTCCT | TATCAACTGGGCTCGGGTCA | |
| mouse-*5Htr2a* | TCTGTGCCGTCTGGATTTACC | TCCTGTAGCCCGAAGACTGG | |
| mouse-*5Htr2b* | GTTAATTTCAATAGGCATCGCCA | AGCCAGTGACCCAAAGACCAT | |
| mouse-*5Htr2c* | CGGTTCAATTCGCGGACTAAGG | GGTCATTGAGCACGCAGGTAGT | |
| mouse-*5Htr3a* | CACACTCCTTCTGGGATACTCAG | GATGGTCTCAGCGAGGCTTATC | |
| mouse-*5Htr3b* | GTGTCCTCTACTTACCACATCCG | GTCCACGAGCATCAGGAAGATG | |
| mouse-*5Htr7* | CTGCCTCCATCACCTTACCTC | GTTTCACCACGCCATTCAGG | |
